# Territorial inequalities in the food environment of Brazilian metropolitan regions

**DOI:** 10.1590/1980-549720260040

**Published:** 2026-08-17

**Authors:** Laura Luciano Scaciota, Claudio Makoto Kanai, Marcos Anderson Lucas da Silva, Renata Bertazzi Levy

**Affiliations:** I Universidade de São Paulo, Center for Epidemiological Research in Nutrition and Health – São Paulo (SP), Brazil.; II Universidade de São Paulo, Medical School, Department of Preventive Medicine – São Paulo (SP), Brazil; III Universidade de São Paulo, School of Public Health, Department of Nutrition – São Paulo (SP), Brazil.; IV University of Salamanca, Institute of Biomedical Research of Salamanca – Salamanca, Spain.

**Keywords:** Food environment, Socioeconomic disparities in health, Food supply, Access to healthy foods

## Abstract

**Objective:**

To describe the retail food environment of Brazilian metropolitan regions using intramunicipal socioeconomic indicators, applying the Locais–NOVA classification.

**Methods:**

Data from the 2023 Annual Social Information Report were used, with Human Development Units (HDUs) as the unit of analysis. Food establishments were geocoded and assigned to HDUs. The Locais-NOVA methodology was applied, which classifies food acquisition outlets according to the degree of food processing and proposes five food environment indicators. The percentage of each indicator was calculated for HDUs and stratified into tertiles of the Municipal Human Development Index (MHDI), considering the distribution of HDUs, Brazilian macroregions, and capitals *versus* non-capitals. A food environment healthiness indicator was created to measure the competition between healthy and unhealthy outlets and was used for spatial description through maps.

**Results:**

A total of 8,977 HDUs across 24 metropolitan regions were assessed, with a mean MHDI of 0.76 and an average of 42.8 food establishments per HDU. Most food outlets were classified as sources of ultra-processed foods (64.9%), with a higher proportion in the first MHDI tertile (67.5%) compared with the third tertile (63.1%). A lower presence of outlets sourcing fresh and minimally processed foods was observed in the first MHDI tertile compared with the third (25.8 vs. 32.3%). The Southeast showed the highest percentage of outlets sourcing processed foods (17.3%). Non-capital concentrated a higher proportion of outlets sourcing processed foods compared with capitals.

**Conclusion:**

A high prevalence of outlets sourcing ultra-processed foods was observed, particularly in territories with worse socioeconomic conditions.

## INTRODUCTION

Access to food is influenced by the area in which people live, both by the presence and concentration of outlets and by the types of food sold ^1,2^. The number and type of outlets tend to reflect the socioeconomic conditions of the areas in which they are located, revealing patterns of inequality in the territorial distribution of food availability^3^.

Environments with a higher density of outlets that predominantly sell ultra-processed foods are associated with increased purchasing and consumption of those foods^4,5^. The intrinsic characteristics of ultra-processed foods, combined with their commercial practices, produce harmful effects on consumers’ health^6^ and contribute to their widespread presence in food environments^7^.

Identifying the most socioeconomically vulnerable areas and analyzing the distribution of food outlets in those spaces is essential to inform urban planning and to guide public policies addressing the social and commercial determinants of health.

While international studies analyze the food environment at a national scale^8,9^, Brazilian scientific production remains concentrated in limited geographic areas and focused on the Southeast region^10-13^. Additionally, studies of food environments using secondary data employ a wide range of methods to determine which food outlets are considered healthy^14-17^. This heterogeneity persists even among studies based on the NOVA classification^18-21^.

Recently, an innovative methodology was proposed for categorizing food outlets. Called Locais-Nova, it incorporates the recommendations of the Dietary Guidelines for the Brazilian Population and is applicable to all regions of Brazil, allowing comparisons across different contexts^22^. The objective of this study was to describe the retail food environment of Brazilian metropolitan regions according to intramunicipal socioeconomic indicators, using the Locais-Nova classification.

## METHODS

### Data source

This is a descriptive ecological study carried out in all Brazilian Metropolitan Regions (MRs) and Integrated Regions of Economic Development (RIDE) with intramunicipal socioeconomic indicators. Data from the Atlas of Human Development in Brazilian Metropolitan Regions, the 2023 Annual List of Social Information (Relação Anual de Informações Sociais, RAIS 2023)^23^ and the Brazilian Postal Service^24^ were used.

#### Atlas of Human Development in Brazilian Metropolitan Regions

The Atlas of Human Development is a platform produced by the Institute of Applied Economic Research (Ipea), the United Nations Development Programme (UNDP), and the João Pinheiro Foundation^25^. The Atlas created Human Development Units (HDUs), spatial divisions formed by grouping census tracts with similar socioeconomic characteristics, ensuring greater socioeconomic homogeneity within the territory. This grouping allows for the assessment of inequalities within the same municipality. The HDUs represent the smallest Brazilian territorial units for which the Municipal Human Development Index (MHDI) is calculated and were the unit of analysis for this study^26^.

The MHDI is based on the global Human Development Index (HDI) and adapted to the Brazilian context. The index is the geometric mean of three subindices: longevity (life expectancy at birth), education (adult educational attainment and school flow of the young population), and income (per capita income). The MHDI ranges from 0 to 1, with values closer to 1 indicating higher human development in the HDU^27^.

The Atlas provides the HDU spatial mesh (shapefile format) for 24 Brazilian Metropolitan Regions (MRs) and Integrated Regions of Economic Development (RIDEs) (Supplementary Table 1).

#### Annual List of Social Information

RAIS is a socioeconomic information report requested by the Ministry of Labor and Employment from employers and legal entities^23^. Establishment owners annually complete a declaration with information such as the establishment name, number of employees, state, municipality, postal code (CEP), and the National Classification of Economic Activities (CNAE)^28^.

CNAE is the national standardization instrument for economic activity codes for commercial establishments, enabling the identification of different types of businesses.

#### Brazilian Postal Service

The National Address Directory is a database from the Brazilian Postal Service that contains complete addresses for Brazil, including information such as state, city, neighborhood, street name, CEP, and geographic coordinates. The database was acquired in 2021 in .csv format^24^.

### Data treatment and analysis

The study was carried out in eight stages. In the first stage, commercial establishments of interest were selected from the RAIS database. Included CNAEs were: supermarkets (including hypermarkets), minimarkets, bakeries (production and retail), dairy retailers, candy stores, butcher shops, fishmarkets, produce markets/greengrocers, convenience stores, frozen and ready-to-eat food vendors (delicatessens and delivery), restaurants, snack bars, and bars (with and without entertainment). CNAEs for canteens were not considered, as they are private spaces accessed only by patrons of institutions such as schools, hospitals, and workplaces; street food vendors were excluded because they are poorly represented in RAIS due to informality; and beverage retailers, such as wholesalers and distributors, were excluded because they are not classified under Locais-Nova.

The second stage involved matching the Brazilian Postal Service geographic coordinates to the commercial establishments using postal codes.

In the third stage, establishments were georeferenced in QGIS using the coordinates obtained in the previous stage, creating a vector point layer. The mean percentage loss due to georeferencing issues, such as invalid coordinates, was 1.1% (Supplementary Table 2).

The fourth stage involved linking HDU information to commercial establishments using the "join by location" feature, calculating the number of each type of establishment in each HDU, and calculating the area of the HDUs in km².

In the fifth stage, establishments were grouped according to indicators proposed by the Locais-Nova methodology, which is based on the recommendations of the Dietary Guidelines for the Brazilian Population.

The system created 5 indicators:

1. Food outlets that should be the basis of the diet (sources of unprocessed or minimally processed foods and culinary ingredients; hereafter referred to as *unprocessed* source outlets);
2. Food outlets identified only as sources of foods that should be the basis of the diet (sources exclusively of unprocessed or minimally processed foods and culinary ingredients; hereafter referred to as only unprocessed source outlets);
3. Food outlets that are sources of foods that should be limited (sources of processed foods; hereafter referred to as processed source outlets);
4. Food outlets that are sources of foods that should be avoided (sources of ultra-processed foods; hereafter referred to as ultra-processed source outlets); and
5. Food outlets identified only as sources of foods that should be avoided (sources exclusively of ultra-processed foods; hereafter referred to as only-ultra-processed source outlets).

To capture regional differences, Locais-Nova provides region-specific indicators for each macro-region, which were adopted in this study (Supplementary Box 1). Details on the development of the indicators can be found in Silva et al.^22^.

The sixth stage was the descriptive analysis of the data. Percentages of each indicator were calculated for the HDUs and stratified by MHDI terciles (higher tercile = higher MHDI), considering the overall distribution of HDUs, by macro-region, and between state capitals and non-capitals. Analyses were performed using Stata/SE 16.1^27^.

The seventh stage consisted of creating the food environment healthiness indicator, defined as the ratio between the number of "only-unprocessed source outlets" (numerator) and the number of "ultra-processed source outlets" (denominator), according to the Locais-Nova classification. Because the same establishment can simultaneously be a source of unprocessed foods and of ultra-processed products, the numerator includes only outlets that do not sell ultra-processed foods, while the denominator includes all outlets that are sources of those products.

The healthiness indicator was based on the recommendation to avoid consumption of ultra-processed foods and on the understanding that their availability represents an obstacle to adequate and healthy eating^29^. Higher values indicate a greater relative predominance of outlets offering exclusively unprocessed foods compared with those offering ultra-processed foods, characterizing environments more favorable to healthy eating.

Finally, in the eighth stage, the spatial distribution of the data across the HDUs of the 24 MRs and RIDEs was described. Bivariate maps were produced based on the food environment healthiness indicator (ratio of only-unprocessed source outlets to ultra-processed source outlets) and MHDI terciles, calculated separately for each metropolitan region, considering the distribution of HDUs within each region.

The food environment healthiness indicator is divided into five categories, each with a corresponding color:

1. HDUs with ultra-processed source outlets and no only *unprocessed* source outlets (dark pink);
2. HDUs with a predominance of ultra-processed source outlets over only-*unprocessed* source outlets (light pink);
3. HDUs with equal proportions of ultra-processed source outlets and only-*unprocessed* source outlets (white);
4. HDUs with a predominance of only-unprocessed source outlets over ultra-processed source outlets (light blue);
5. HDUs with only-unprocessed source outlets and no ultra-processed source outlets (dark blue).

The higher the MHDI, the brighter the color used.

Spatial analyses were conducted in QGIS version 3.22.14 using the SIRGAS 2000 Coordinate Reference System. The Albers projection was used to calculate HDU areas^30^.

#### Data availability statement:

The data supporting the findings of this study are publicly available and can be accessed via the Atlas Brasil portal and the Ministry of Labor. The combined dataset is not publicly available because it results from integration of databases carried out by the authors.

## RESULTS

A total of 8,977 HDUs were evaluated across the 24 metropolitan regions, with a mean MHDI of 0.76. HDUs with lower MHDI had a higher mean population (8,811.3) and larger territorial area (25.8 km²), whereas HDUs with higher MHDI had a smaller population (7,446.6) and smaller area (2.4 km²) but a higher mean number of food establishments (75.7).

Most acquisition sites are sources of ultra-processed foods (64.9%). There is approximately a 5% difference between the 1st (67.5% and 58.6%) and 3rd (63.1% and 53.9%) MHDI terciles for ultra-processed source outlets and only-ultra-processed source outlets, respectively. The presence of only-unprocessed source outlets is 34% higher in HDUs with the highest MHDI (29.6%) compared with those with the lowest MHDI (22.1%) (Table 1).

**Table 1:** Characteristics of the Human Development Units and percentage of establishments by Locais-Nova categories, by MHDI terciles, in metropolitan regions with intramunicipal socioeconomic indicators: 2023.

		Municipal Human Development Index level			Total
		MHDI 1	MHDI 2	MHDI 3	
		0.666 (0.500–0.715)*	0.758 (0.716–0.800)*	0.864 (0.801–0.970)*	0.762 (0.500–0.970)*
Characteristics of HDUs					
	Number of HDUs (%)	3000 (33.4)	3030 (33.8)	2947 (32.8)	8977
	Mean population per HDU (95%CI)	8811.3 (8452.9–9169.7)	8192.7 (7891.2–8494.2)	7446.6 (7141.0–7752.2)	8154.5 (7967.7; 8341.3)
	Mean HDU area (95%CI)	25.8 (15.6–35.9)	4.9 (3.3–6.5)	2.4 (2.1–2.8)	11.1 (7.6–14.5)
	Mean number of total food establishments (95%CI)	19.0 (17.9–20.1)	34.3 (32.6–35.9)	75.7 (70.8–80.6)	42.8 (40.9–44.6)
Mean percentage of establishments by Locais-Nova categories					
	Mean percentage of only-unprocessed source outlets† (95%CI)	22.1 (21.3–22.9)	25.4 (24.8–26.1)	29.6 (28.9–30.2)	25.7 (25.2–26.1)
	Mean percentage of unprocessed source outlets† (95%CI)	25.8 (25.0–26.7)	29.2 (28.5–29.8)	32.3 (31.6–32.9)	29.1 (28.6–29.5)
	Mean percentage of processed-source outlets† (95%CI)	15.5 (14.8–16.2)	16.0 (15.4–16.6)	13.8 (13.3–14.3)	15.1 (14.8–15.5)
	Mean percentage of ultra-processed source outlets†(95%CI)	67.5 (66.6–68.5)	64.2 (63.4–64.9)	63.1 (62.4–63.8)	64.9 (64.5–65.4)
	Mean percentage of only-ultra-processed source outlets†(95%CI)	58.6 (57.6–59.6)	54.8 (54.0–55.5)	53.9 (53.2–54.6)	55.8 (55.3–56.2)

* Mean MHDI (range of variation)

† Percentage rate=number of source outlets*100/ total number of food retail establishments

HDU: Human Development Units; MHDI: Municipal Human Development Index; CI: confidence interval.

Source: Elaborated by the author.

The Southeast contains the largest number of HDUs (5,574) and the highest population density (population/area) and total food-establishment density (total establishments/area). In all regions, population density and food outlet density increase from the first to the third MHDI tercile. The North has the fewest HDUs (395) and the largest disparities between terciles: in the third MHDI tercile, food outlet density is 208 times higher and population density is 40 times higher than in the first MHDI tercile. State capitals generally have smaller mean area, larger populations, and more food establishments than non-capitals. Comparing MHDI extremes, capitals show a greater difference in population density (5.4 times in the 3rd MHDI tercile vs. the 1st, compared with 2.1 times in non-capitals), while non-capitals show a greater difference in food-establishment density (41 times in the 3rd MHDI tercile vs. the 1st, compared with 25 times in capitals) (Table 2). Moreover, in all regions the mean percentage of outlets that are sources of ultra-processed foods is higher than that of outlets that are sources of *unprocessed* foods (Table 3).

**Table 2:** Characteristics of Human Development Units by Brazilian macro-regions, state capitals and non-capitals, by Municipal Human Development Index terciles; 2023.

			MHDI 1	MHDI 2	MHDI 3	Total
			n (95%CI)	n (95%CI)	n (95%CI)	n (95%CI)
Number of HDUs						
	Region					
		South	341	322	327	990
		Southeast	1,897	1,852	1,825	5,574
		Midwest	280	210	205	623
		Northeast	466	472	457	1,395
		North	134	130	131	395
	Situation					
		Capital	1,507	1,496	1,483	4,486
		Non-capital	1,508	1,542	1,441	4,491
Mean population in the HDUs						
	Region					
		South	6311.9 (5528.4–7095.3)	7458.7 (6592.0–8325.3)	7496.8 (6529.5–8464.2)	7076.3 (6571.2–7581.3)
		Southeast	7020.5 (6673.6–7367.3)	8152.4 (7259.3–7982.1)	7620.7 (7259.3–7982.1)	7593.1 (7388.6–7797.6)
		Midwest	12570.3 (10607.4–14533.2)	8704.1 (7514.7–9893.5)	8204.4 (6410.0–9998.7)	9830.5 (8851.0–10809.9)
		Northeast	13943.5 (12801.4–15085.5)	9268.9 (8363.9–10174.0)	7230.5 (6453.3–8007.8)	10162.7 (9592.2–10733.1)
		North	14019.8 (11481.2–16558.4)	10629.3 (8481.3–12777.3)	4714.3 (3653.8–5774.7)	9817.8 (8591.5–11044.1)
	Situation					
		Capital	10152.3 (9523.0–10781.5)	8008.7 (7578.7–8438.7)	8613.6 (8098.4–9128.8)	8928.8 (8620.7–9236.9)
		Non-capital	7956.0(7565.7–8346.4)	8059.8; 7686.3–8433.2)	6265.4; 5947.6–6583.2)	7449.2 (7238.1–7660.3)
Mean area of HDUs per km^2^						
	Region					
		South	25.6 (10.2–40.9)	6.2 (4.7–7.8)	2.2 (1.8–2.6)	11.6 (6.2–16.9)
		Southeast	8.9 (5.5–12.2)	4.8 (2.4–7.3)	2.1 (1.8–2.4)	5.3 (3.9–6.7)
		Midwest	66.4 (23.9–108.8)	8.5 (5.0–12.0)	6.9 (3.3–10.6)	27.3 (12.9–41.7)
		Northeast	39.8 (24.6–55.0)	1.6 (1.4–1.8)	1.7 (1.3–2.1)	14.4 (9.2–19.6)
		North	156.4 (-45.7–358.5)	1.4 (1.1–1.8)	1.3 (0.8–1.8)	54.0 (-14.8–122.7)
	Situation					
		Capital	14.0 (-4.3–28.5)	2.4 (1.9–3.0)	2.2 (1.7–2.7)	6.3 (1.4–11.1)
		Non-capital	36.0 (21.9–50.2)	8.3 (5.3–11.4)	2.9 (2.5–3.3)	15.9 (11.0–20.8)
Mean number of total food establishments						
	Region					
		South	17.4 (14.5–20.4)	39.0 (33.7–44.2)	87.5 (72.4–102.6)	47.6 (41.9–53.3)
		Southeast	14.0 (13.0–15.0)	32.4 (30.5–34.4)	75.5 (69.6–81.4)	40.3 (38.1–42.4)
		Midwest	43.1 (34.5–51.7)	48.5 (40.5–56.6)	99.4 (68.6–130.3)	63.5 (52.4–74.5)
		Northeast	29.2 (25.2; 33.2)	34.3 (30.1–38.6)	67.0 (56.3–77.6)	43.3 (39.2–47.4)
		North	22.5 (17.2–27.7)	30.2 (23.6–36.7)	40.7 (2.3–52.2)	31.1 (26.3–35.8)
	Situation					
		Capital	23.3 (21.4–25.1)	39.0 (26.2–41.8)	93.4 (84.8–102.0)	51.7 (48.5–57.9)
		Non-capital	17.0 (15.4–18.5)	29.2 (27.4–31.1)	56.4 (52.1–60.7)	33.8 (32.1–35.5)

MHDI: Municipal Human Development Index; CI: confidence interval; HDU: Human Development Units.

Source: Elaborated by the author.

**Table 3:** Mean percentage of food outlets in Locais-Nova categories relative to total food outlets, by Brazilian macro-regions and Municipal Human Development Index terciles; 2023.

		Mean percentage of only-unprocessed source outlets* % (95%CI)	Mean percentage of unprocessed source outlets* % (95%CI)	Mean percentage of processed-source outlets* % (95%CI)	Mean percentage of ultra-processed source outlets* % (95%CI)	Mean percentage of only-ultra-processed source outlets* % (95%CI)
South						
	Total	23.2 (22.0–24.5)	27.8 (26.5–29.1)	9.9 (9.0–10.8)	75.8 (74.5–77.1)	61.8 (60.4–63.2)
	1st MHDI tercile	15.0 (12.8–17.1)†	20.8 (18.4–23.1)†	10.2 (8.4–12.1)	83.9 (81.6–86.2)†	68.7 (66.0–71.3)†
	2nd MHDI tercile	24.0 (21.0–26.0)	28.5 (26.5–30.5)	11.2 (9.6–12.7)	75.1 (73.1–77.1)	59.6 (57.5–61.8)
	3rd MHDI tercile	31.1 (29.0–33.1)	34.3 (32.3–36.3)	8.2 (7.0–9.4)	68.1 (66.0–70.2)	56.7 (54.6–58.9)
Southeast						
	Total	25.0 (24.5–25.5)	28.6 (28.1–29.2)	17.3 (16.9–17.8)	63.2 (62.6–63.8)	54.1 (53.4–54.7)
	1st MHDI tercile	22.4 (21.4–23.5)†	26.3 (25.2–27.4)†	17.9 (16.9–18.8)‡	64.8 (63.6–66.0)‡	55.8 (54.6–57.1)†
	2nd MHDI tercile	24.8 (24.0–25.6)	28.9 (28.0–29.8)	17.9 (17.1–18.6)	62.6 (61.7–63.5)	53.2 (52.3–54.2)
	3rd MHDI tercile	27.9 (27.1–28.7)	30.7 (29.9–31.5)	16.3 (15.6–16.9)	62.3 (61.4–63.1)	53.0 (52.1–53.9)
Midwest						
	Total	27.6 (26.3–28.9)	35.2 (33.9–36.6)	10.9 (10.0–11.7)	72.4 (71.1–73.7)	53.9 (52.5–55.3)
	1st MHDI tercile	22.2 (20.0–24.3)†	33.0 (30.5–35.4)‡	10.6 (9.1–12.2)	77.8 (75.7–80.0)†	56.4 (53.8–59.0)
	2nd MHDI tercile	29.1 (26.9–31.5)	36.3 (34.1–38.6)	11.3 (10.1–12.6)	70.8 (68.5–73.1)	52.3 (50.0–54.6)
	3rd MHDI tercile	31.5 (29.5–33.5)	36.3 (34.2–38.5)	10.6 (8.9–12.3)	68.5 (66.5–70.5)	53.1 (50.9–55.3)
Northeast						
	Total	28.9 (27.9–30.0)	28.9 (27.9–30.0)	12.7 (11.9–13.5)	58.3 (57.2–59.5)	58.4 (57.2–59.5)
	1st MHDI tercile	25.2 (23.3–27.2)†	25.2 (23.3–27.2)†	13.6 (12.2–15.4)†	61.1 (59.0–63.3)‡	61.1 (59.0–63.2)‡
	2nd MHDI tercile	27.9 (26.1–29.7)	27.9 (26.1–29.7)	14.5 (13.1–16.0)	57.6 (55.5–59.7)	57.6 (55.5–59.7)
	3rd MHDI tercile	33.8 (32.0–35.6)	33.8 (32.0–35.6)	9.9 (8.8–11.0)	56.3 (54.4–58.2)	56.3 (54.4–58.2)
North						
	Total	26.7 (24.5–28.8)	29.4 (27.1–31.6)	12.2 (10.8–13.6)	73.4 (71.2–75.5)	58.4 (56.1–60.8)
	1st MHDI tercile	22.3 (18.2–26.5)†	24.7 (20.4–28.9)†	13.0 (10.1–16.0)	77.7 (73.5–81.8)†	62.3 (57.7–65.9)‡
	2nd MHDI tercile	25.6 (22.4–28.8)	29.2 (25.9–32.5)	11.8 (9.9–13.7)	74.4 (71.2–77.6)	59.0 (55.6–62.5)
	3rd MHDI tercile	32.1 (28.4–35.9)	34.4 (30.6–38.1)	11.7 (9.3–14.1)	67.9 (64.1–71.6)	53.9 (50.1–57.7)

* Percentage rate=number of source outlets*100/total number of food retail establishments

† p for trend <0.01

‡ p for trend <0.05.

MHDI: Municipal Human Development Index; CI: confidence interval; HDU: Human Development Units.

Source: Elaborated by the author.

In the South, differences between MHDI terciles are marked. The percentage of only-unprocessed source outlets in the first MHDI tercile is half that of the third. The Southeast has the highest percentage of processed-source outlets (17.3%), while the Center-West stands out for the highest proportion of unprocessed source outlets (35.2%) and the Northeast for the lowest percentage of ultra-processed source outlets (58.3%). In the North, differences between MHDI terciles are relatively homogeneous, except for processed-source outlets, which do not vary.

State capitals concentrate 4% more only-ultra-processed source outlets and 6% more only-*in-natura* source outlets. The largest disparity between terciles occurs for only-in-natura source outlets, favoring HDUs with higher MHDI (8.4% variation in capitals and 6.5% in non-capitals) (Table 4).

**Table 4:** Mean percentage of food outlets in Locais-Nova categories relative to total food outlets, by Human Development Units, state capitals and non-capitals, and Municipal Human Development Index terciles; 2023.

		Mean percentage of only-unprocessed source outlets* % (95%CI)	Mean percentage of unprocessed source outlets* % (95%CI)	Mean percentage of processed-source outlets* % (95%CI)	Mean percentage of ultra-processed source outlets* % (95%CI)	Mean percentage of only-ultra-processed source outlets* % (95%CI)
Capital						
	Total	26.4 (25.8–27.0)	28.9 (28.3–29.5)	14.1 (13.6–14.6)	65.3 (64.7–65.9)	56.9 (56.2–57.5)
	1st MHDI tercile	22.4 (21.2–23.5)†	25.0 (23.8–26.2)†	14.9 (13.9–15.9)†	67.8 (66.5–69.1)†	60.1 (58.7–61.4)†
	2nd MHDI tercile	26.1 (25.2–27.0)	29.0 (28.1–30.0)	14.6 (13.9–15.4)	65.2 (64.1–66.2)	56.3 (55.3–57.4)
	3rd MHDI tercile	30.8 (29.9–31.7)	32.9 (32.0–33.8)	12.8 (12.1–13.5)	62.9 (61.9–63.8)	54.2 (53.2–55.2)
Non-capital						
	Total	24.9 (24.4–25.5)	29.2 (28.6–29.8)	16.1 (15.6–16.6)	64.6 (63.9–65.2)	54.6 (54.0–55.3)
	1st MHDI tercile	21.8 (20.7–22.9)†	26.3 (25.1–27.5)†	15.8 (14.8–16.7)	67.5 (66.2–68.9)†	57.9 (56.5–59.3)†
	2nd MHDI tercile	24.9 (24.0–25.8)	29.6 (28.6–30.5)	17.0 (16.2–17.9)	63.6 (62.6–64.7)	53.3 (52.2–54.4)
	3rd MHDI tercile	28.3 (27.3–29.2)	31.9 (30.9–32.8)	15.5 (14.7–16.2)	62.5 (61.5–63.5)	52.6 (51.6–53.6)

CI: confidence interval; MHDI: Municipal Human Development Index.

* Percentage rate=number of source outlets*100/total number of food retail establishments

† p for trend <0.01.

Source: Elaborated by the author.

**Figure 1:**
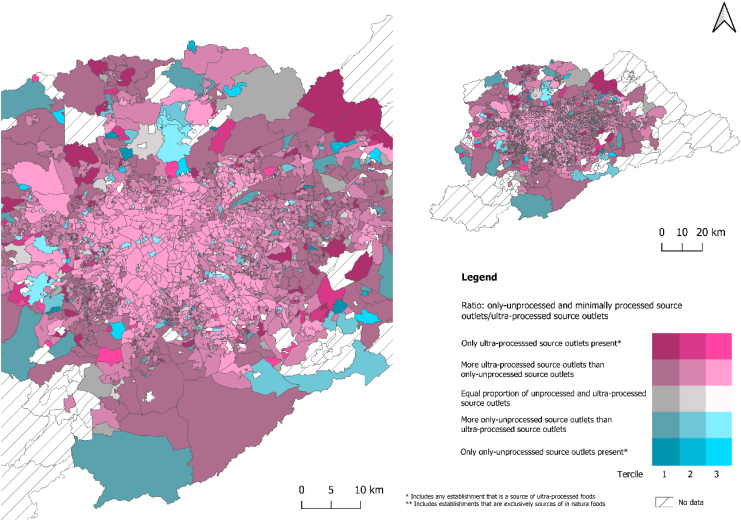
Spatial distribution of the food environment healthiness indicator (ratio of outlets offering only unprocessed and minimally processed foods to outlets offering ultra-processed foods) in the São Paulo Metropolitan Region.

Source: Elaborated by the author.

The maps show the spatial distribution of the food environment healthiness indicator (ratio of ultra-processed source outlets to only-unprocessed source outlets). In all metropolitan regions and integrated regions of economic development, pink predominates, indicating a predominance of ultra-processed source outlets in the HDUs. Bright pink, representing greater predominance of these outlets in the third MHDI tercile, is concentrated in urban centers and coastal areas of the metropolitan regions. The map of the São Paulo Metropolitan Region, which has the largest population and number of food establishments, is shown in Figure 1. The maps for the other regions are available in the supplementary material (Supplementary Figures 1-23). Few HDUs show a balanced proportion between the two categories. In Baixada Santista, Belém, Belo Horizonte, Maceió, Rio de Janeiro, São Paulo, Sorocaba, Vale do Paraíba, Vale do Rio Cuiabá, and Vitória, the proportion of HDUs with a predominance of ultra-processed source outlets increases with higher MHDI terciles. In the other regions the opposite pattern is observed, except in the Federal District and Porto Alegre, where neither pattern was observed.

## DISCUSSION

Socioeconomic differences were observed in the spatial distribution of food establishments. Lower-income areas have lower population concentration and fewer available establishments, both in territorial density and relative to the HDU population.

In countries such as China, Singapore, and the United Kingdom, lower-socioeconomic areas have higher densities of food establishments^31,32^. In Mexico and Brazil, socioeconomically disadvantaged areas show lower concentrations of these establishments^33-36^, likely due to differing socioeconomic contexts and development patterns compared with high-income countries.

In the Global North, higher population density in less developed areas attracts establishments due to greater demand^31^. In developing countries, poor urban infrastructure in vulnerable areas favors the expansion of the informal market, which may explain the lower concentration of establishments in the first MHDI tercile. These regions were more affected by the COVID-19 pandemic, with closures of establishments that did not reopen^36,37^. In Rio Grande do Sul, minimarkets showed the largest decline between 2010 and 2022^36^, being more frequent in socioeconomically disadvantaged areas^38^. In the United Kingdom, there was an increase in food delivery establishments in disadvantaged areas and a decrease in advantaged areas in the post-pandemic period^39^.

Outlets selling ultra-processed foods account for nearly two-thirds of the total and are concentrated in the first MHDI tercile. Outlets selling unprocessed foods represent less than one-third of all food establishments and are concentrated in the third MHDI tercile. Thus, residents of less advantaged areas face greater barriers to following the recommendations of the Dietary Guidelines for the Brazilian Population, whose golden rule is "prefer *unprocessed* and minimally processed foods and culinary preparations over ultra-processed foods"^29^.

The Southeast shows the smallest differences between MHDI terciles, indicating similar exposures to food environments despite different social contexts. It also has the highest mean percentage of processed-source outlets. In this region, bakeries are classified as processed-source outlets, whereas in other regions they are also classified as ultra-processed-source outlets. In São Paulo, the metropolitan region with the largest number of HDUs, bakeries are central to local food culture, which may explain the higher percentage of processed-source outlets in the Southeast.

In the South, 83.9% of establishments in the first MHDI tercile are sources of ultra-processed foods, indicating greater exposure to unhealthy food environments in less advantaged areas compared with other regions and MHDI terciles. A study estimating the caloric share of ultra-processed foods in Brazilian municipalities found higher mean consumption in the South^40^. Although conducted in different periods and using different data sources, the findings converge and demonstrate consistency between the availability and consumption of ultra-processed foods in the region.

The Dietary Guidelines for the Brazilian Population recommend avoiding purchases from outlets that sell exclusively ultra-processed foods as a strategy to overcome supply-related barriers^29^. The presence of ultra-processed foods in the environment can hinder healthier food choices, even when unprocessed and minimally processed foods are available.

This dynamic can be explained by the commercial determinants of health, understood as strategies used by commercial actors to expand markets and influence behaviors and health conditions^7^. In the case of ultra-processed foods, these include marketing practices and retail organization that favor their availability and visibility in food environments^41,42^, giving them a competitive advantage over unprocessed and minimally processed foods. Supermarkets, which simultaneously supply ultra-processed and unprocessed foods, exemplify this process. Small producers and family farmers face barriers to placing their products in these outlets due to contracts with long payment terms, consignment sales, and requirements for standardized size and shape of fruits and vegetables. Conversely, large ultra-processed food corporations have the resources to secure better shelf space^41^, one of the main facilitators of retail sales^43-46^. This advantage is reflected in purchasing patterns, with ultra-processed foods accounting for a larger share of supermarket purchases^47^.

Contrary to expectations, no large differences were observed between capitals and non-capitals for any category of food outlets. This result may be explained by the selection of municipalities located in metropolitan regions, which may share characteristics with capitals, such as average population, degree of urbanization, and income.

The use of maps makes it possible to objectively identify the most vulnerable territories that should be prioritized in public policies aimed at transforming the food environment, making them an important strategic tool to support public management.

Creating healthy food environments is essential to promote healthy eating and requires intersectoral actions between the health and food supply sectors. Programs, policies, and interventions should support healthier choices, such as incentivizing the opening of outlets that sell only unprocessed and minimally processed foods in areas with low availability of these items^48^.

The study has limitations inherent to the ecological design. The presence of food outlets does not necessarily reflect individuals’ actual purchasing behavior. Results may be affected by the Modifiable Areal Unit Problem (MAUP), related to biases from aggregating spatial analysis units^49^, especially in larger HDUs where different distribution patterns of establishments may coexist but be represented by a single average.

Another limitation concerns the use of multiple secondary data sources, which may vary in coverage, timeliness, and data quality. Still, these sources allowed analysis of establishment distribution across a wide territorial coverage and the identification of spatial patterns in the food environment across different socioeconomic contexts.

Self-reporting in RAIS by establishment owners may produce errors in the classification of economic activity. Moreover, due to the nature of the database, only formal food establishments could be assessed. Data on open-air markets and other food security and nutrition facilities are not available for all evaluated cities. Nonetheless, using a secondary database containing information on all formal food establishments is what made a study of this magnitude possible.

Georeferencing based on the postal code centroid is another limitation; however, the postal code (CEP) is the most precise location data available for the establishments and is a widely used measure in food-environment studies^50^.

This study assessed the availability of food establishments without considering the time and mode of travel required to access them or the prices charged. The analysis was restricted to metropolitan regions, limiting the generalizability of the findings to other contexts.

Finally, there is a temporal mismatch between RAIS 2023 data and the socioeconomic information from the 2010 Demographic Census. The Human Development Units (HDUs) have socioeconomic indicators calculated only for that Census, with no more recent estimates available at the same territorial disaggregation.

Among the study's strengths is its scope: it is the most extensive description of the food environment ever conducted in Brazil, covering 24 metropolitan regions across the five macro-regions. The use of the Locais-Nova methodology is another strength, as it is grounded in the country's actual food consumption and acquisition realities.

The findings provide support for the development of public supply and healthy eating policies and underscore the need for strategies that consider regional inequalities in food availability. They point to actions to support the opening and maintenance of outlets that sell only unprocessed and minimally processed foods, as well as regulation of outlets that sell ultra-processed foods. These measures are especially important in areas dominated by ultra-processed foods, which hinder healthy choices.
