# Supplementary material for: Territorial inequalities in the food environment of Brazilian metropolitan regions
Source: Rev Bras Epidemiol. 2026 Aug 17;29:e260040. doi: 10.1590/1980-549720260040 (PMC13480622; doi:10.1590/1980-549720260040)
Supplement: Supplementary file 1 [file 1980-5497-rbepid-29-e260040-Suppl01-pt.pdf]

Tabela Suplementar 1. Número de Unidades de Desenvolvimento Humano e IDHM médio nas Regiões Metropolitanas e Regiões Integradas de Desenvolvimento Econômico estudadas.

Região Metropolitana / Regiões Integradas de Desenvolvimento Econômico	Número de UDH	IDHM médio (95%IC)
Baixada Santista (SP)	203	0,77 (0,76; 0,78)
Belém (PA)	192	0,76 (0,74; 0,77)
Belo Horizonte (MG)	439	0,75 (0,74; 0,76)
Campinas (SP)	364	0,79 (0,78; 0,80)
Curitiba (PR)	251	0,76 (0,75; 0,77)
Distrito Federal e entorno (DF e MG)	290	0,78 (0,77; 0,79)
Florianópolis (SC)	223	0,81 (0,80; 0,82)
Fortaleza (CE)	280	0,73 (0,72; 0,74)
Goiânia (GO)	223	0,79 (0,77; 0,80)
Maceió (AL)	111	0,74 (0,72; 0,76)
Manaus (AM)	203	0,75 (0,73; 0,76)
Natal (RN)	94	0,75 (0,73; 0,77)
Petrolina-Juazeiro (BA e PE)	64	0,70 (0,68; 0,73)
Porto Alegre (RS)	516	0,75 (0,74; 0,76)
Recife (PE)	373	0,73 (0,72; 0,74)
Rio de Janeiro (RJ)	1643	0,75 (0,75; 0,76)
Salvador (BA)	271	0,76 (0,75; 0,77)
São Luís (MA)	90	0,78 (0,77; 0,80)
São Paulo (SP)	2202	0,77 (0,77; 0,77)
Sorocaba (SP)	203	0,77 (0,77; 0,78)
Teresina e Timon (PI e MA)	112	0,72 (0,70; 0,73)
Vale do Paraíba e Litoral Norte (SP)	254	0,78 (0,77; 0,79)
Vale do Rio Cuiabá (MT)	123	0,78 (0,76; 0,79)
Vitória (ES)	253	0,76 (0,75; 0,77)

Tabela Suplementar 2. Perdas decorrentes do georreferenciamento da Relação Anual de Informações Sociais de 2023, segundo Região Metropolitana ou Região Integrada de Desenvolvimento Econômico.

Relação Anual de Informações Sociais (RAIS) 2023			
Região metropolitana e Região Integrada de Desenvolvimento Econômico	Número de estabelecimentos de alimentos		% perdas
	Antes do georreferenciamento	Após o georreferenciamento	
Baixada Santista (SP)	8836	8789	0,5
Belém (PA)	5182	5129	1,0
Belo Horizonte (MG)	23545	23035	2,2
Distrito Federal e entorno (DF e MG)	22584	22008	2,6
Campinas (SP)	15969	15837	0,8
Curitiba (PR)	20772	20644	0,6
Florianópolis (SC)	9225	9180	0,5
Fortaleza (CE)	13986	13863	0,9
Goiânia (GO)	12447	12219	1,8
Maceió (AL)	4417	4402	0,3
Manaus (AM)	7090	7072	0,3
Natal (RN)	5417	5353	1,2
Petrolina-Juazeiro (BA e PE)	2257	2223	1,5
Porto Alegre (RS)	17119	16891	1,3
Recife (PE)	12536	12354	1,5
Rio de Janeiro (RJ)	40036	39622	1,0
Salvador (BA)	12770	12692	0,6
São Paulo (SP)	107618	106725	0,8
São Luís (MA)	4481	4409	1,6
Sorocaba (SP)	7918	7837	1,0
Teresina (PI)	4551	4548	0,1
Vale do Paraíba e Litoral Norte (SP)	11220	11095	1,1
Vale do Rio Cuiabá (MT)	4990	4881	2,2
Vitória (ES)	8820	8689	1,5
Total	388075	383786	1,1

Quadro Suplementar 1. Locais de aquisição de alimentos segundo a classificação Locais-Nova, Brasil e Grandes Regiões.

Indicadores	Brasil	Norte	Nordeste	Sudeste	Sul	Centro-Oeste
Locais fonte de alimentos in natura ou minimamente processados e de ingredientes culinários	Supermercados<br>Hortifrutigranjeiros<br>Açougues<br>Ambulantes de alimentos<br>Restaurantes<br>Peixarias	Supermercados<br>Hortifrutigranjeiros<br>Açougues<br>Ambulantes de alimentos<br>Restaurantes<br>Peixarias	Supermercados<br>Hortifrutigranjeiros<br>Açougues<br>Ambulantes de alimentos<br>Restaurantes<br>Peixarias	Supermercados<br>Hortifrutigranjeiros<br>Açougues<br>Ambulantes de alimentos<br>Restaurantes<br>Peixarias	Supermercados<br>Hortifrutigranjeiros<br>Açougues<br>Ambulantes de alimentos<br>Restaurantes<br>Peixarias<br>Cantinas	Supermercados<br>Hortifrutigranjeiros<br>Açougues<br>Ambulantes de alimentos<br>Restaurantes<br>Peixarias
Locais fonte apenas de alimentos in natura ou minimamente processados e de ingredientes culinários	Hortifrutigranjeiros<br>Açougues<br>Ambulantes de alimentos<br>Peixarias<br>Restaurantes	Hortifrutigranjeiros<br>Açougues<br>Ambulantes de alimentos<br>Restaurantes<br>Peixarias	Supermercados<br>Hortifrutigranjeiros<br>Açougues<br>Restaurantes<br>Peixarias	Hortifrutigranjeiros<br>Açougues<br>Restaurantes<br>Peixarias	Hortifrutigranjeiros<br>Açougues<br>Ambulantes de alimentos<br>Peixarias<br>Restaurantes<br>Cantinas	Hortifrutigranjeiros<br>Açougues<br>Restaurantes<br>Peixarias
Locais fonte de alimentos processados	Padarias e confeitarias<br>Bares<br>Lojas de conveniência	Padarias e confeitarias<br>Bares<br>Varejistas de laticínios e frios	Padarias e confeitarias<br>Ambulantes de alimentação<br>Varejistas de laticínios e frios	Padarias e confeitarias<br>Ambulantes de alimentação<br>Bares	Padarias e confeitarias<br>Varejistas de laticínios e frios	Padarias e confeitarias<br>Ambulantes de alimentos<br>Lojas de conveniência

	Varejistas de laticínios e frios<br>Cantinas		Cantinas	Lojas de conveniência<br>Varejistas de laticínios e frios<br>cantinas		Varejistas de laticínios e frios
Locais fonte de alimentos ultraprocessados	Supermercados<br>Minimercados e mercearias<br>Padarias e confeitarias<br>Lanchonetes<br>Bares<br>Lojas de conveniência<br>Bombonieres<br>Cantinas<br>Vendas de alimentos congelados e prontos para consumo<br>Outros	Supermercados<br>Minimercados e mercearias<br>Padarias e confeitarias<br>Lanchonetes<br>Bares<br>Lojas de conveniência<br>Bombonieres<br>Varejista de laticínios e frios<br>Cantinas<br>Vendas de alimentos congelados e prontos para consumo<br>Outros	Minimercados e mercearias<br>Lanchonetes<br>Bares<br>Lojas de conveniência<br>Bombonieres<br>Cantinas<br>Vendas de alimentos congelados e prontos para consumo<br>Outros	Supermercados<br>Minimercados e mercearias<br>Lanchonetes<br>Bares<br>Lojas de conveniência<br>Bombonieres<br>Cantinas<br>Vendas de alimentos congelados e prontos para consumo<br>Outros	Supermercados<br>Minimercados e mercearias<br>Padarias e confeitarias<br>Lanchonetes<br>Bares<br>Lojas de conveniência<br>Bombonieres<br>Vendas de alimentos congelados e prontos para consumo<br>Outros	Supermercados<br>Minimercados e mercearias<br>Padarias e confeitarias<br>Lanchonetes<br>Bares<br>Lojas de conveniência<br>Bombonieres<br>Varejistas de laticínios e frios<br>Cantinas<br>Vendas de alimentos congelados e prontos para consumo<br>Outros

Locais fonte apenas de alimentos ultraprocessados	Minimercados e mercearias<br>Lanchonetes<br>Bombonieres<br>Vendas de alimentos congelados e prontos para consumo<br>Outros	Minimercados e mercearias<br>Lanchonetes<br>Lojas de conveniência<br>Bombonieres<br>Cantinas<br>Vendas de alimentos congelados e prontos para consumo<br>Outros	Minimercados e mercearias<br>Lanchonetes<br>Bares<br>Lojas de conveniência<br>Bombonieres<br>Vendas de alimentos congelados e prontos para consumo<br>Outros	Minimercados e mercearias<br>Lanchonetes<br>Bombonieres<br>Vendas de alimentos congelados e prontos para consumo<br>Outros	Minimercados e mercearias<br>Lanchonetes<br>Bares<br>Bombonieres<br>Vendas de alimentos congelados e prontos para consumo<br>Outros	Minimercados e mercearias<br>Lanchonetes<br>Bares<br>Bombonieres<br>Cantinas<br>Vendas de alimentos congelados e prontos para consumo<br>Outros
