# Supplementary material for: Territorial inequalities in the food environment of Brazilian metropolitan regions
Source: Rev Bras Epidemiol. 2026 Aug 17;29:e260040. doi: 10.1590/1980-549720260040 (PMC13480622; doi:10.1590/1980-549720260040)

Figura Suplementar 1. Distribuição espacial do indicador de saudabilidade do ambiente alimentar (Razão: locais fonte apenas de in natura e minimamente processados e fonte de ultraprocessados) na Região Metropolitana da Baixada Santista.

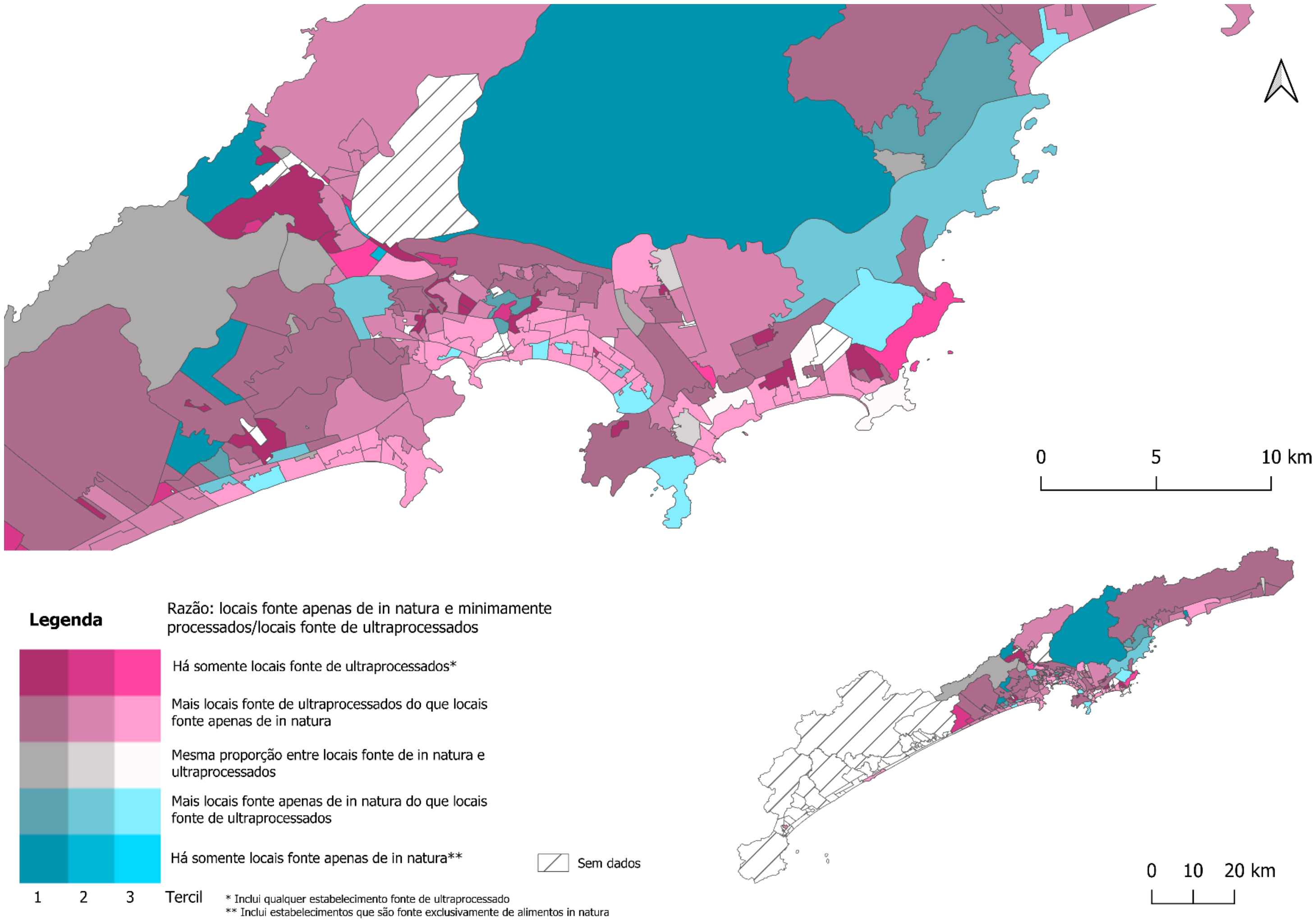

Figura Suplementar 2. Distribuição espacial do indicador de saudabilidade do ambiente alimentar (razão entre locais fonte de apenas de in natura e minimamente processados e fonte de ultraprocessados) na Região Metropolitana de Belém.

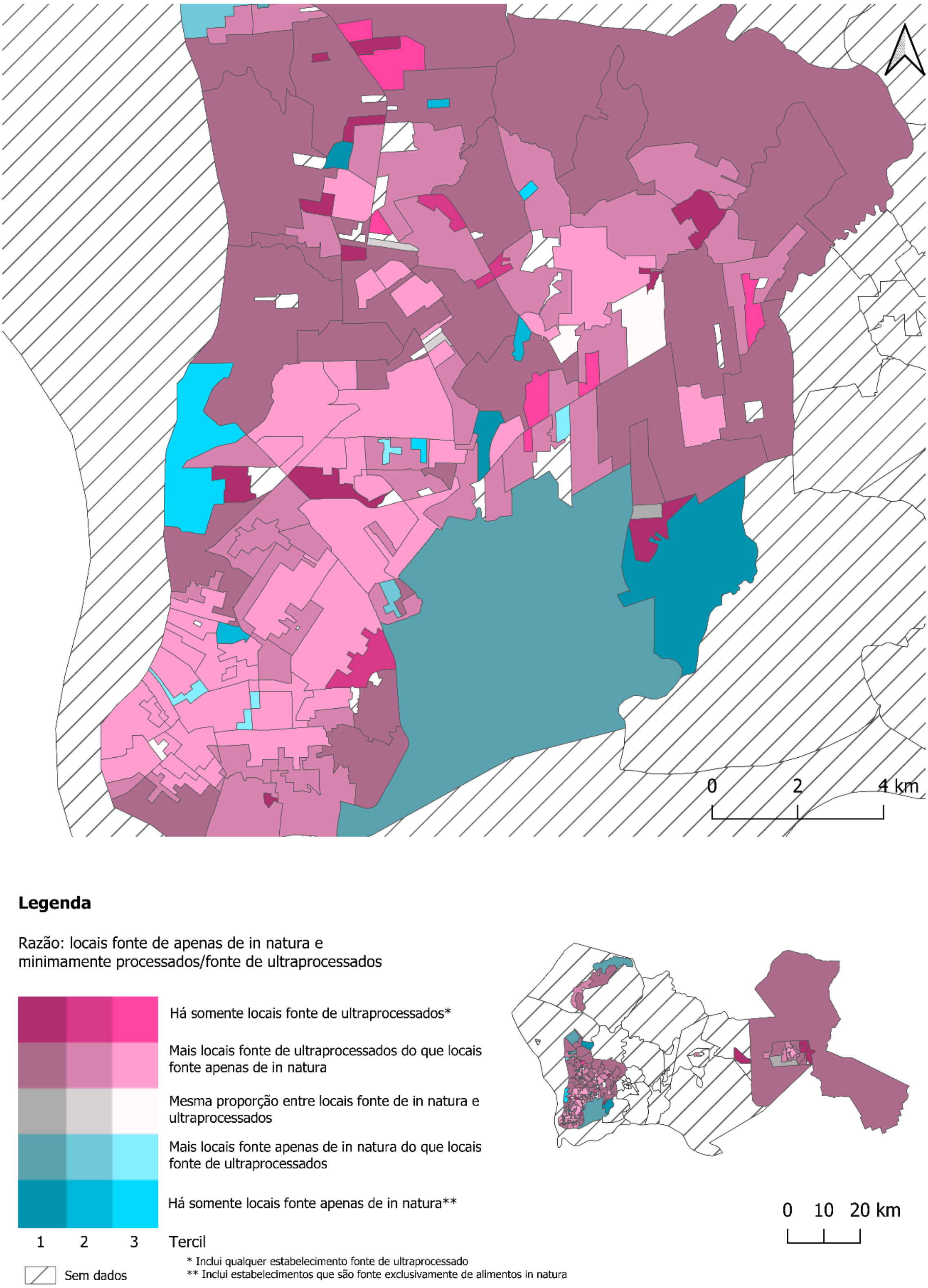

Figura Suplementar 3. Distribuição espacial do indicador de saudabilidade do ambiente alimentar (razão entre locais fonte de apenas de in natura e minimamente processados e fonte de ultraprocessados) na Região Metropolitana de Belo Horizonte.

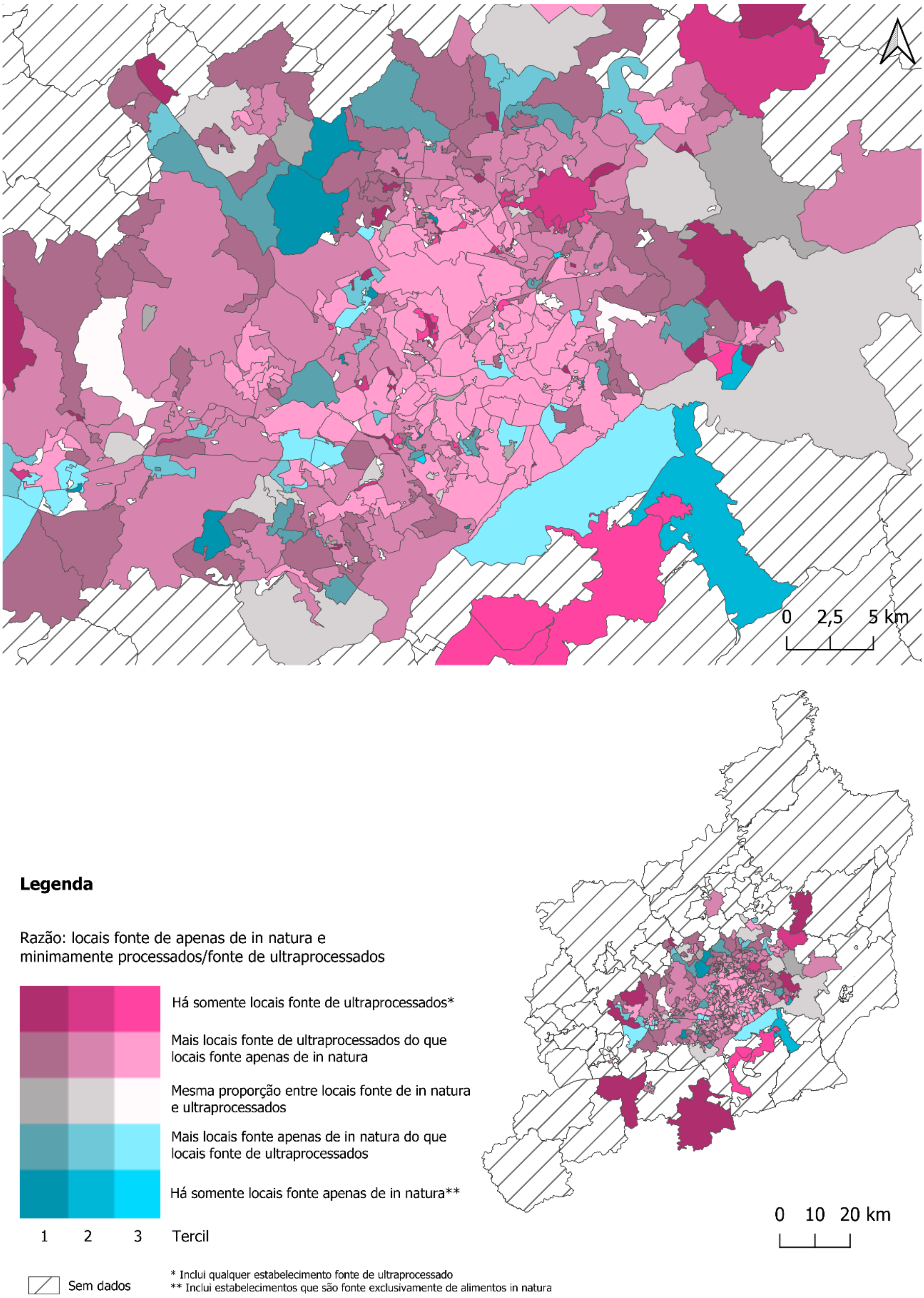

Figura Suplementar 4. Distribuição espacial do indicador de saudabilidade do ambiente alimentar (razão entre locais fonte de apenas de in natura e minimamente processados e fonte de ultraprocessados) na Região Metropolitana de Campinas.

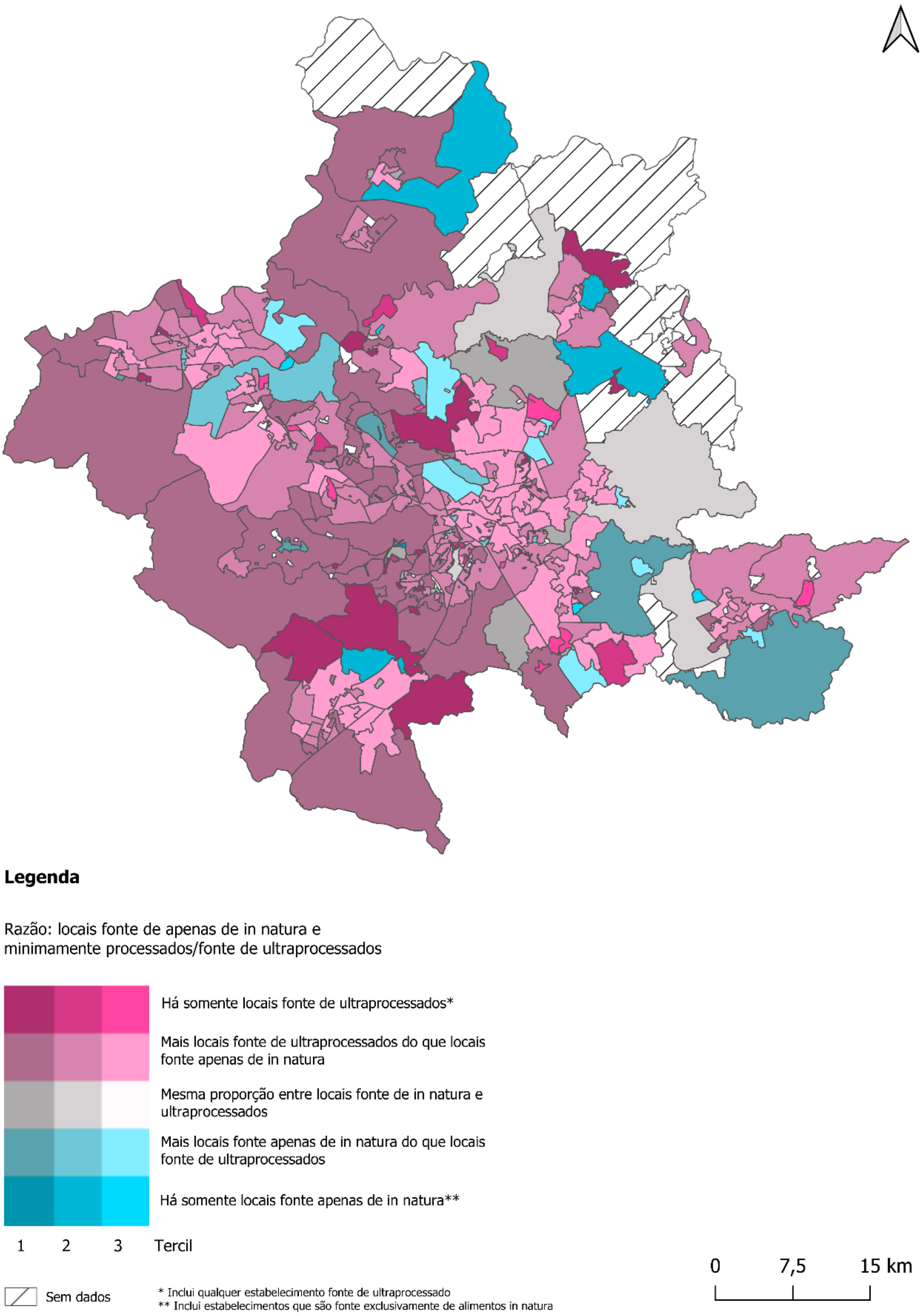

Figura Suplementar 5. Distribuição espacial do indicador de saudabilidade do ambiente alimentar (razão entre locais fonte de apenas de in natura e minimamente processados e fonte de ultraprocessados) na Região Metropolitana de Curitiba.

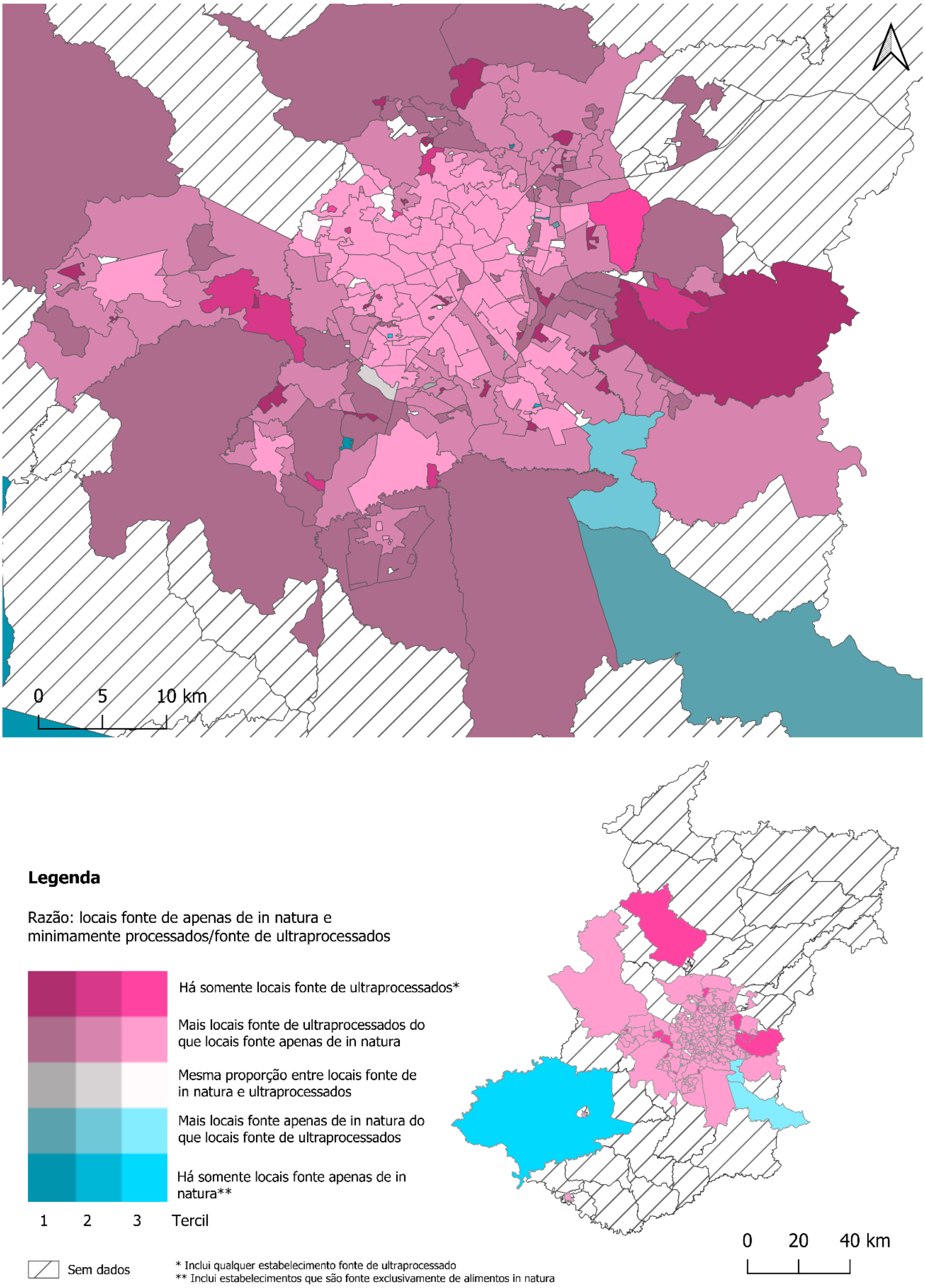

Figura Suplementar 6. Distribuição espacial do indicador de saudabilidade do ambiente alimentar (razão entre locais fonte de apenas de in natura e minimamente processados e fonte de ultraprocessados) na Região Integrada de Desenvolvimento Econômico do Distrito Federal e entorno.

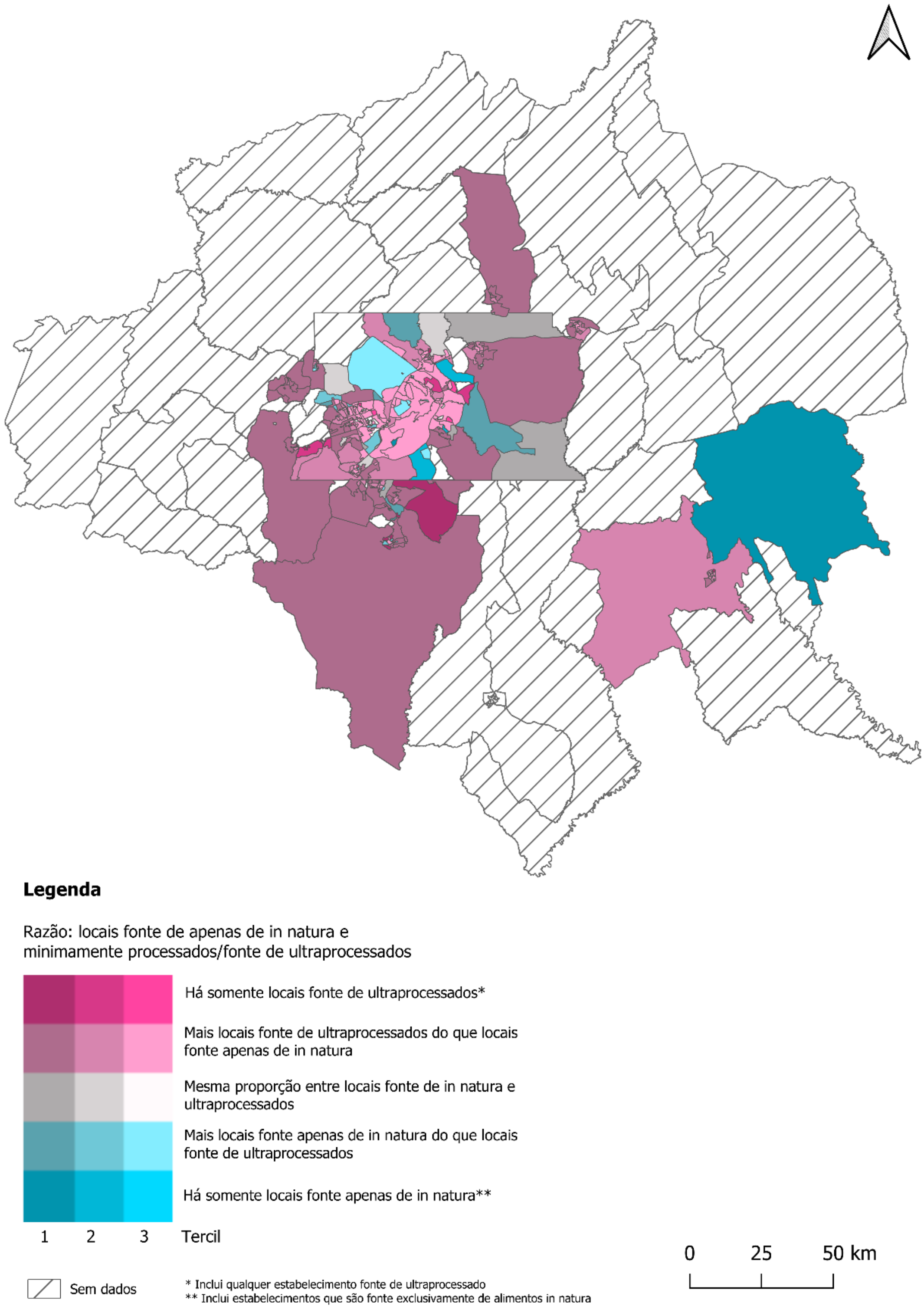

Figura Suplementar 7. Distribuição espacial do indicador de saudabilidade do ambiente alimentar (razão entre locais fonte de apenas de in natura e minimamente processados e fonte de ultraprocessados) na Região Metropolitana de Florianópolis.

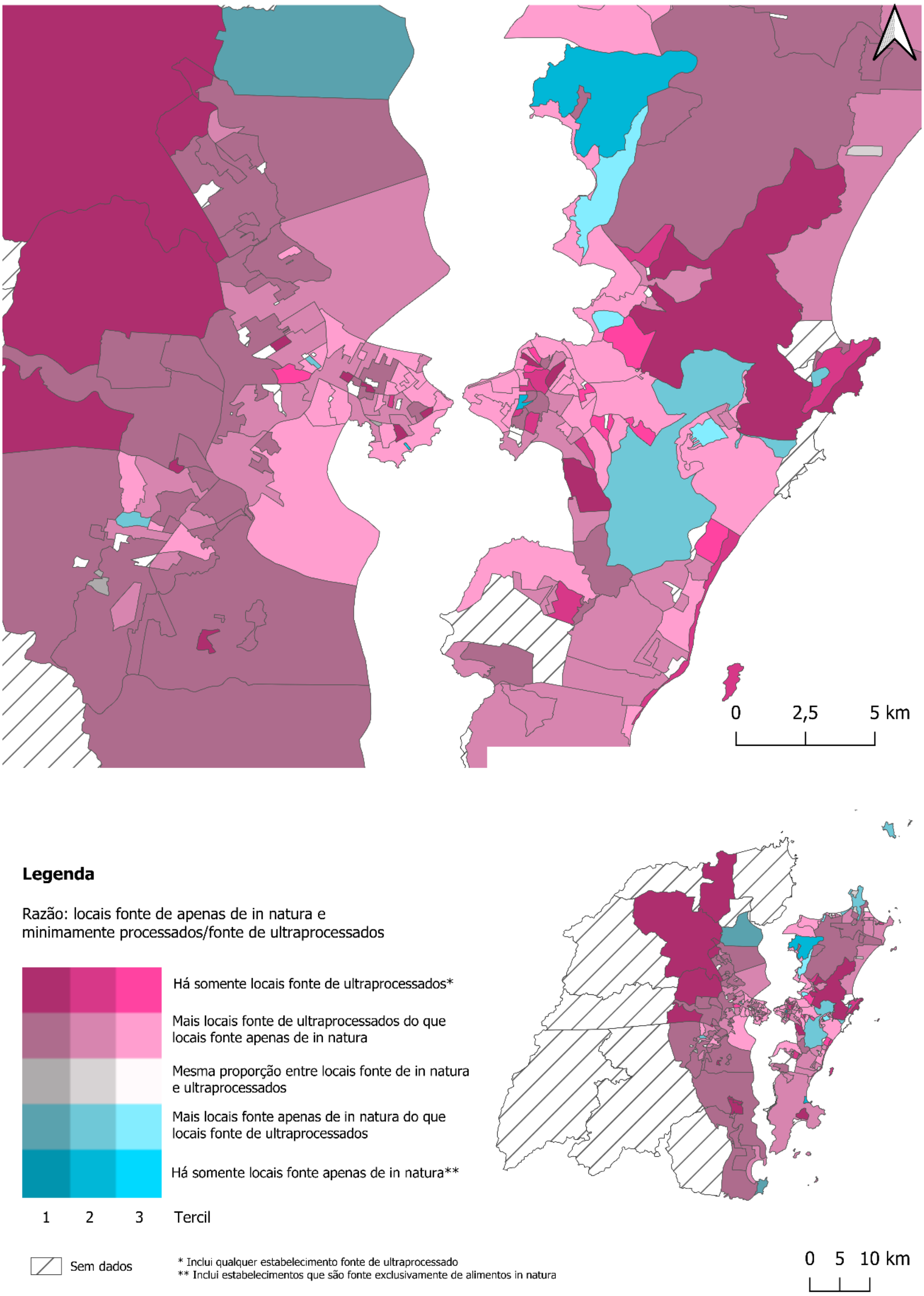

Figura Suplementar 8. Distribuição espacial do indicador de saudabilidade do ambiente alimentar (razão entre locais fonte de apenas de in natura e minimamente processados e fonte de ultraprocessados) na Região Metropolitana de Fortaleza.

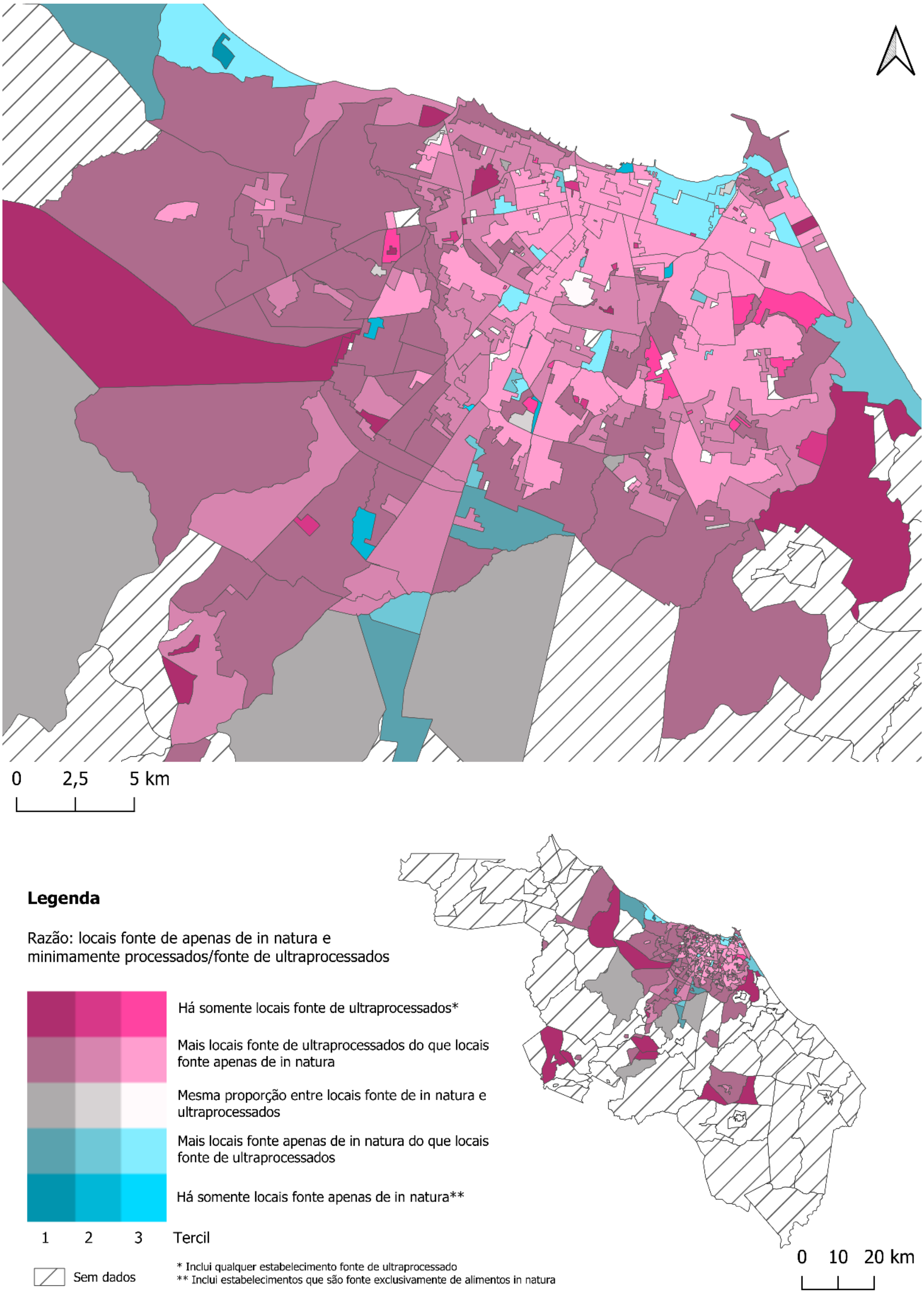

Figura Suplementar 9. Distribuição espacial do indicador de saudabilidade do ambiente alimentar (razão entre locais fonte de apenas de in natura e minimamente processados e fonte de ultraprocessados) na Região Metropolitana de Goiânia.

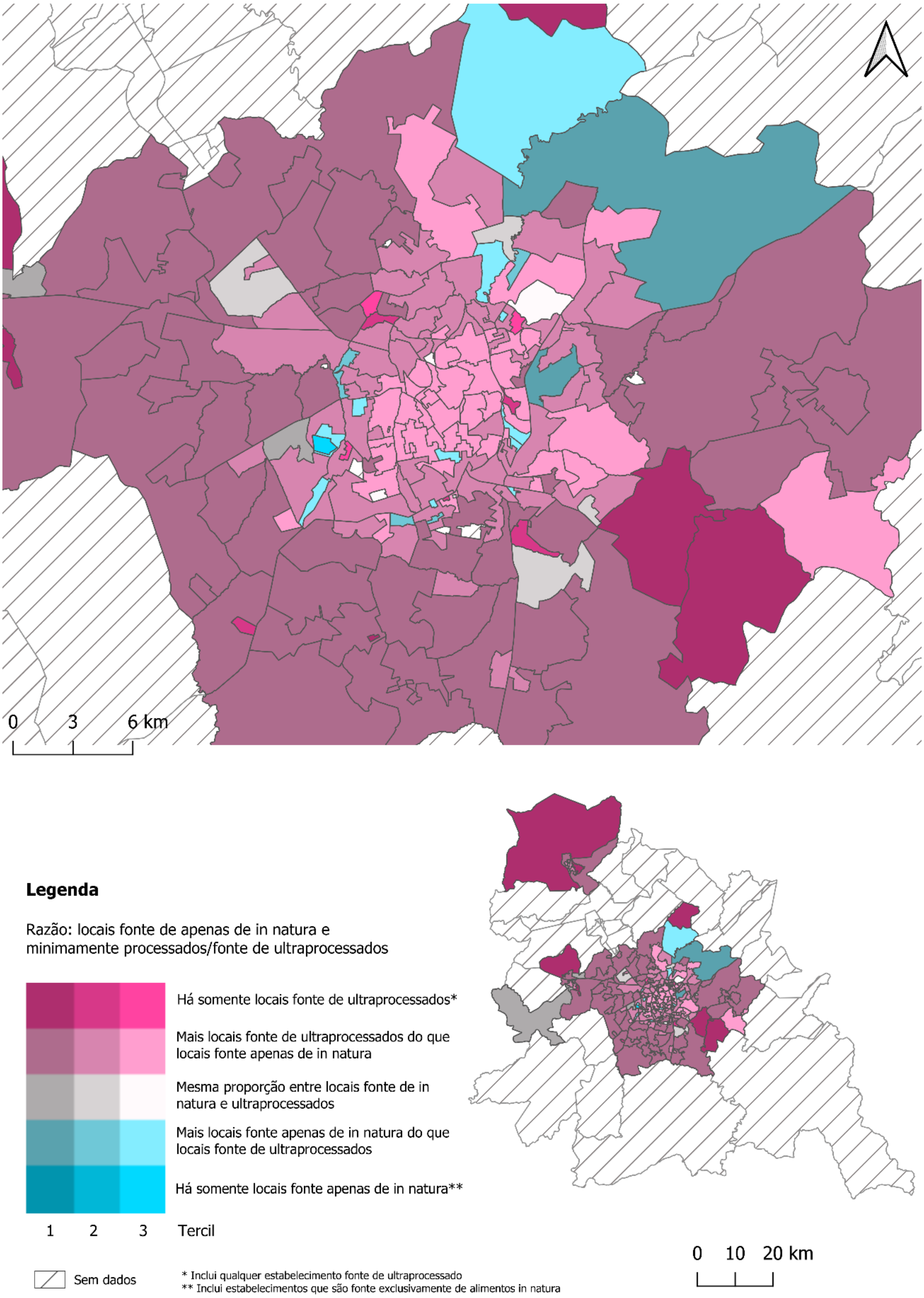

Figura Suplementar 10. Distribuição espacial do indicador de saudabilidade do ambiente alimentar (razão entre locais fonte de apenas de in natura e minimamente processados e fonte de ultraprocessados) na Região Metropolitana de Maceió.

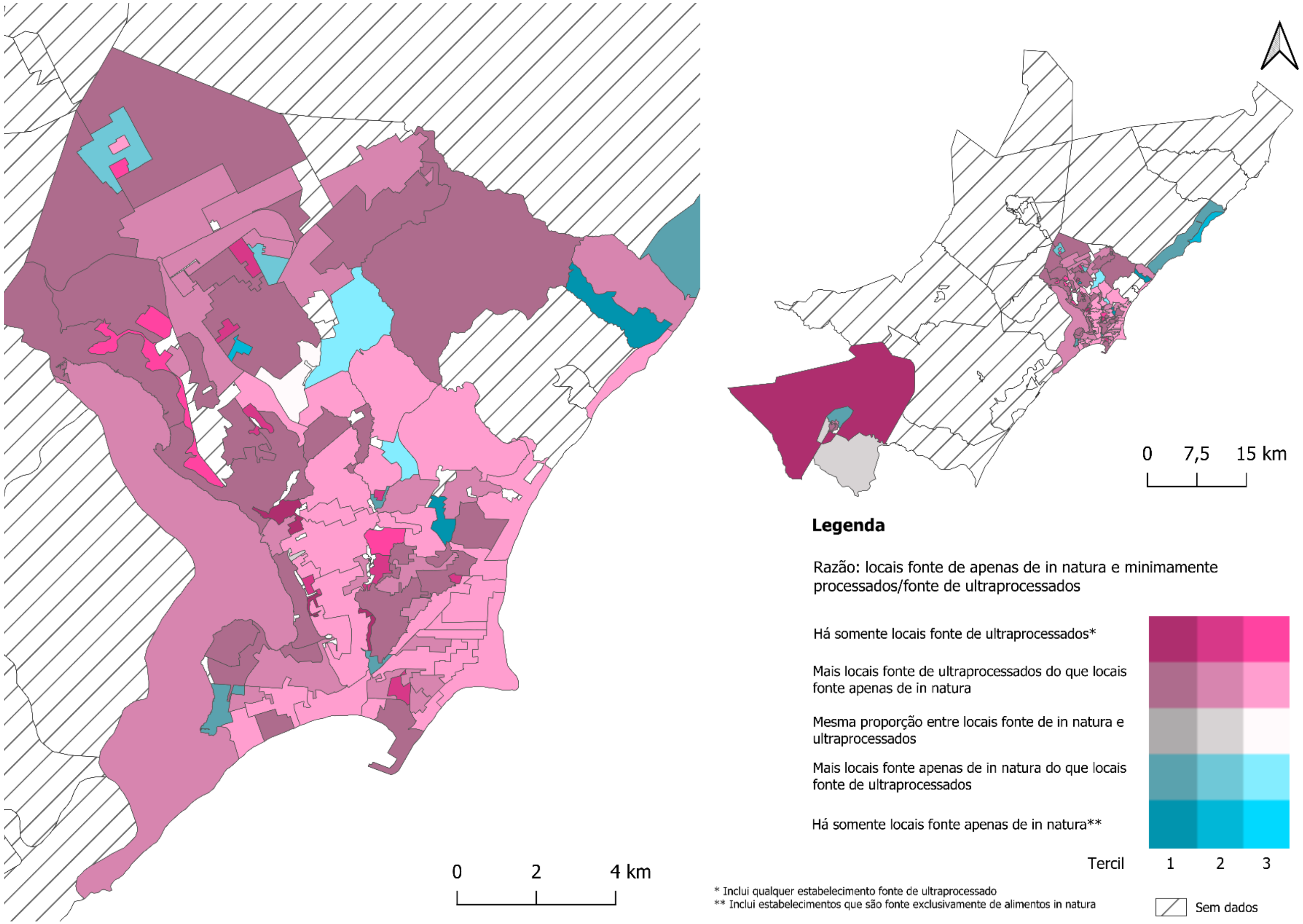

Figura Suplementar 11. Distribuição espacial do indicador de saudabilidade do ambiente alimentar (razão entre locais fonte de apenas de in natura e minimamente processados e fonte de ultraprocessados) na Região Metropolitana de Manaus.

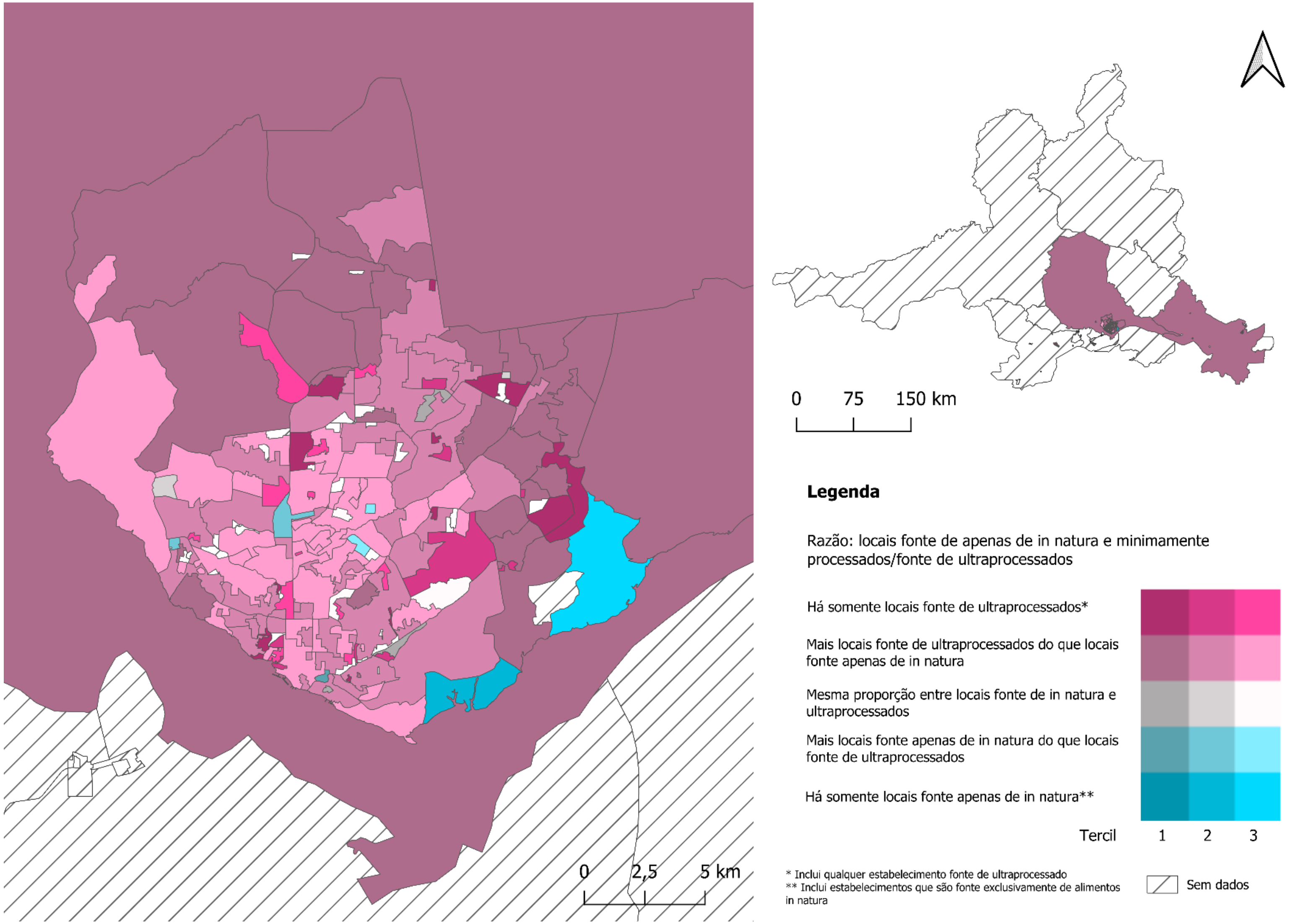

Figura Suplementar 12. Distribuição espacial do indicador de saudabilidade do ambiente alimentar (razão entre locais fonte de apenas de in natura e minimamente processados e fonte de ultraprocessados) na Região Metropolitana de Natal.

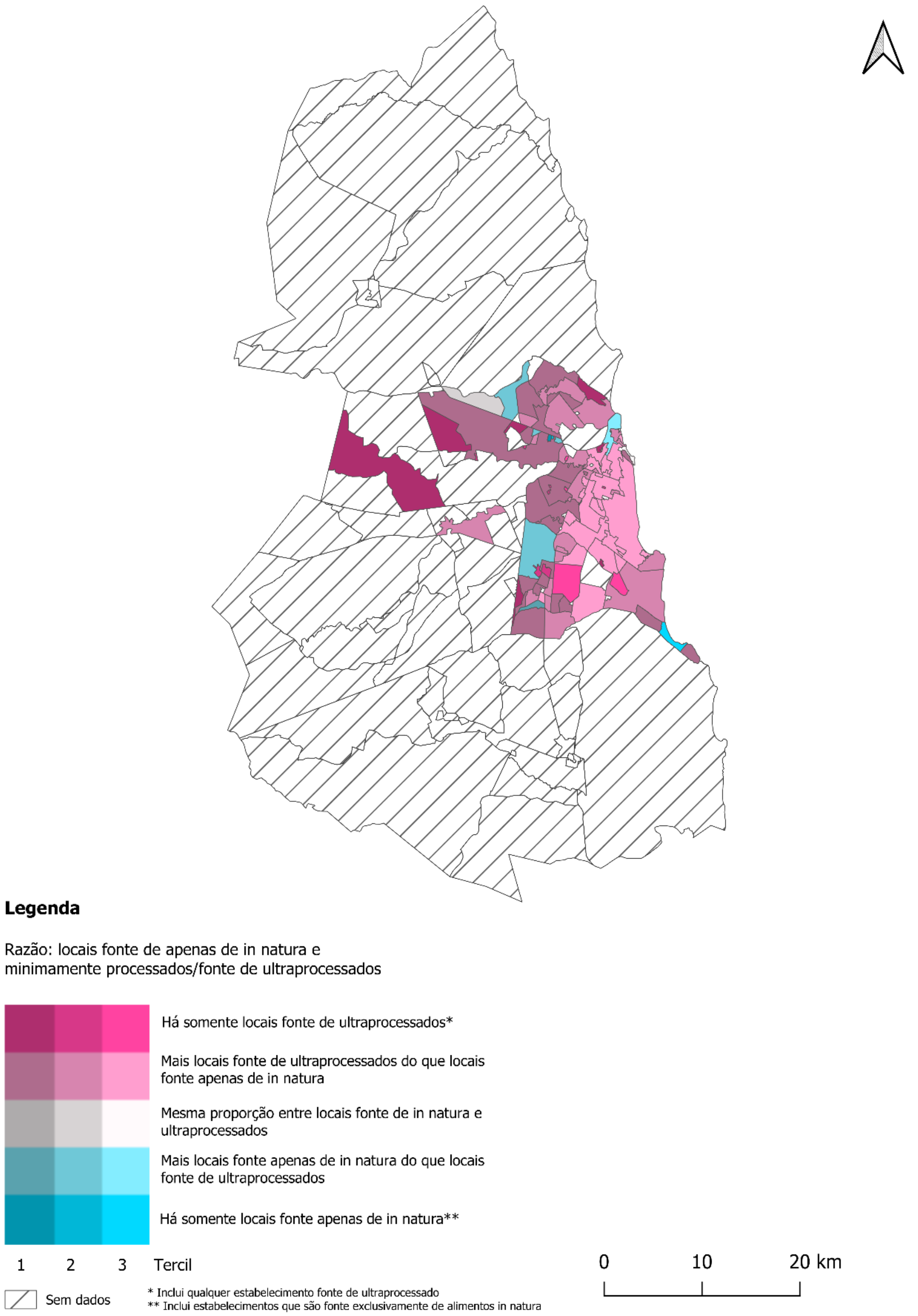

Figura Suplementar 13. Distribuição espacial do indicador de saudabilidade do ambiente alimentar (razão entre locais fonte de apenas de in natura e minimamente processados e fonte de ultraprocessados) na Região Metropolitana de Petrolina-Juazeiro.

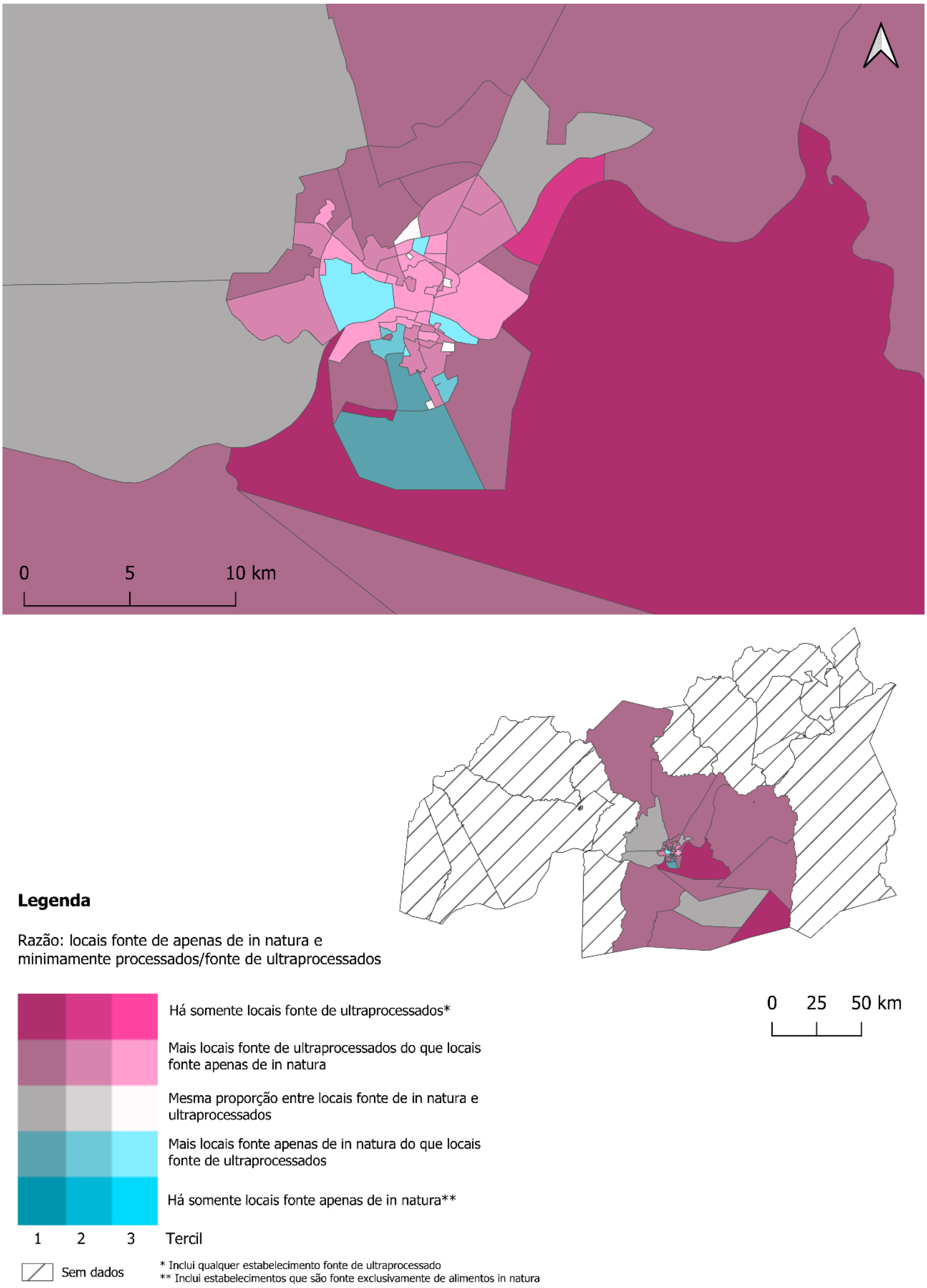

Figura Suplementar 14. Distribuição espacial do indicador de saudabilidade do ambiente alimentar (razão entre locais fonte de apenas de in natura e minimamente processados e fonte de ultraprocessados) na Região Metropolitana de Porto Alegre.

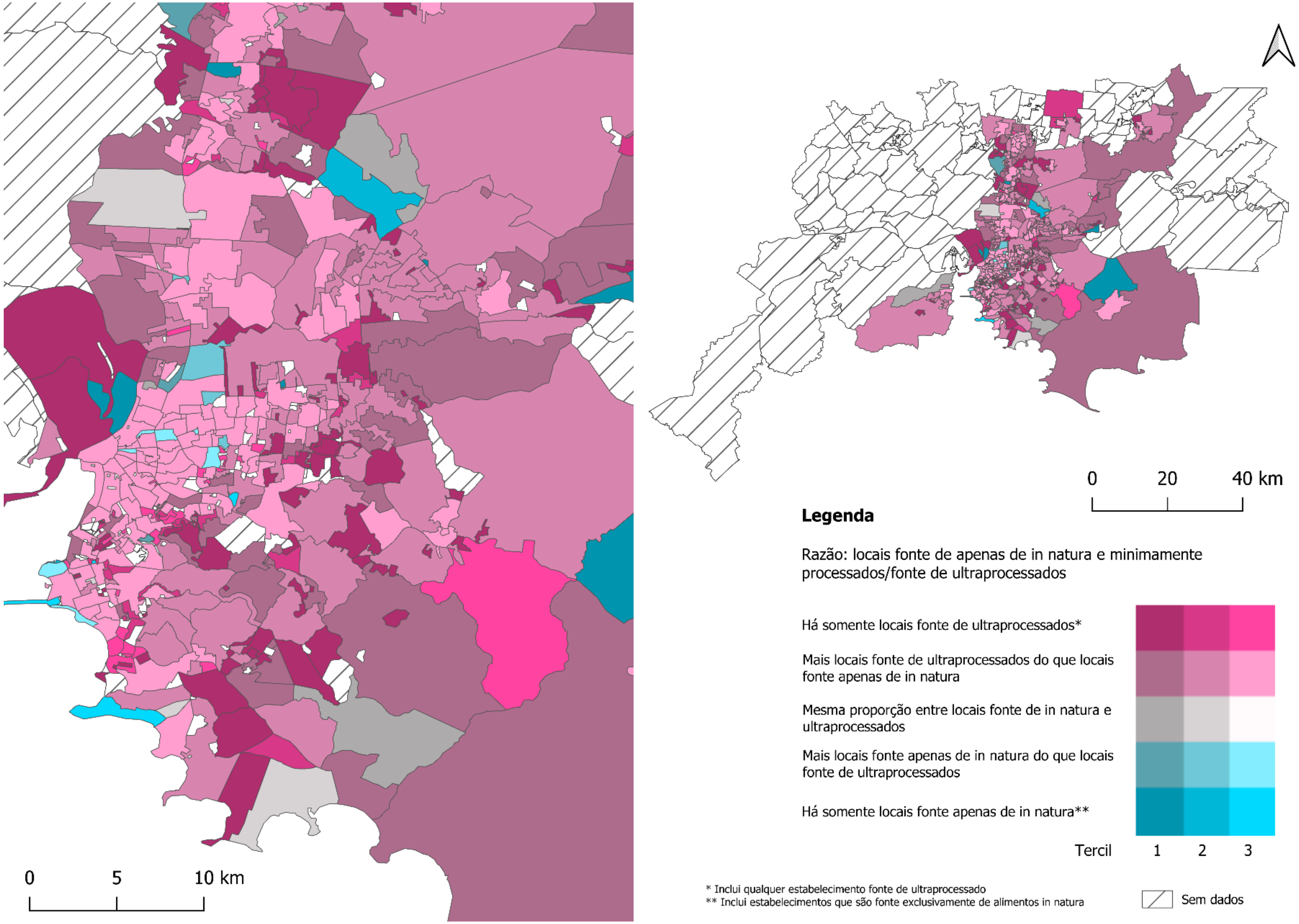

Figura Suplementar 15. Distribuição espacial do indicador de saudabilidade do ambiente alimentar (razão entre locais fonte de apenas de in natura e minimamente processados e fonte de ultraprocessados) na Região Metropolitana de Recife.

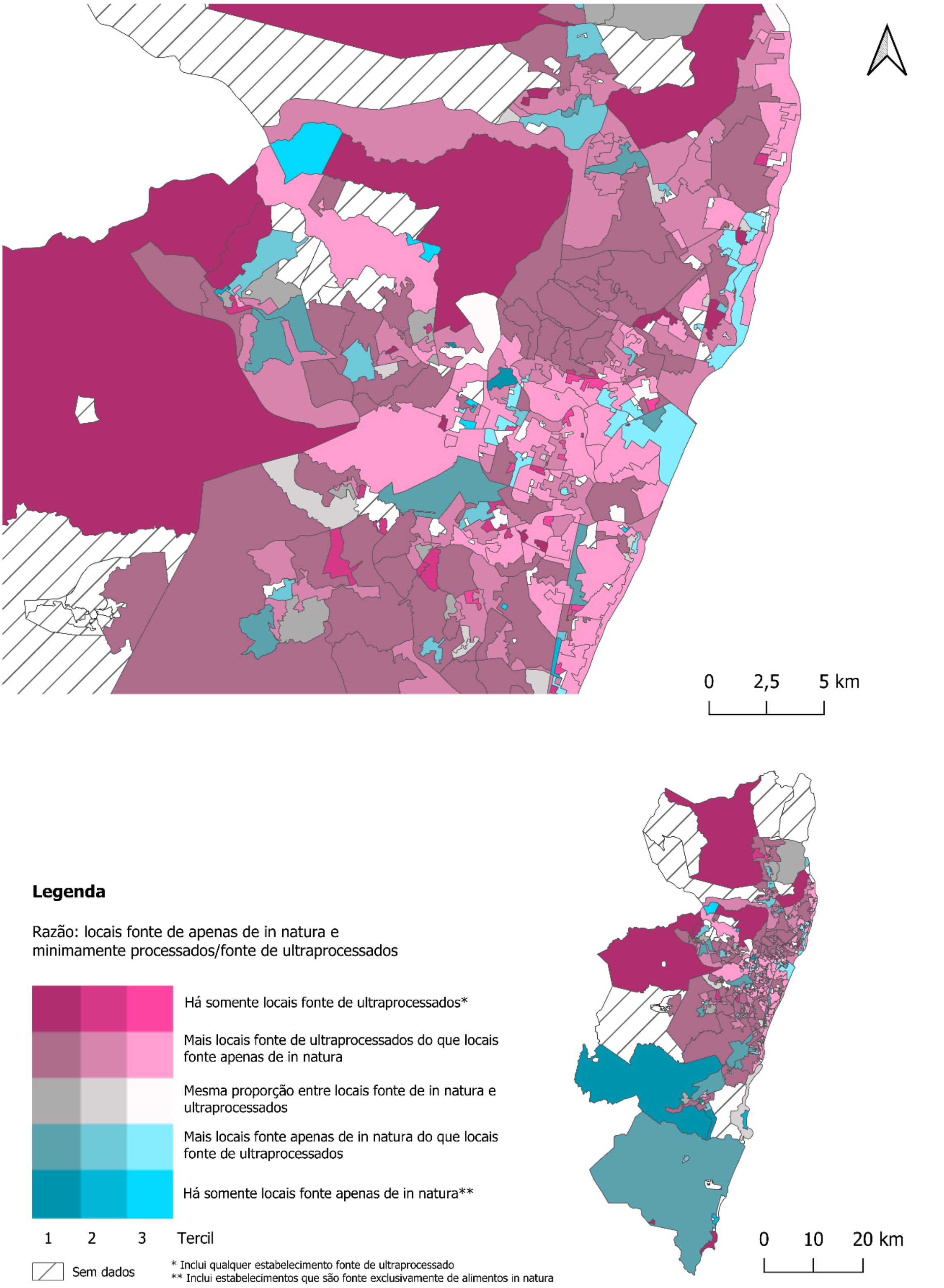

Figura Suplementar 16. Distribuição espacial do indicador de saudabilidade do ambiente alimentar (razão entre locais fonte de apenas de in natura e minimamente processados e fonte de ultraprocessados) na Região Metropolitana do Rio de Janeiro.

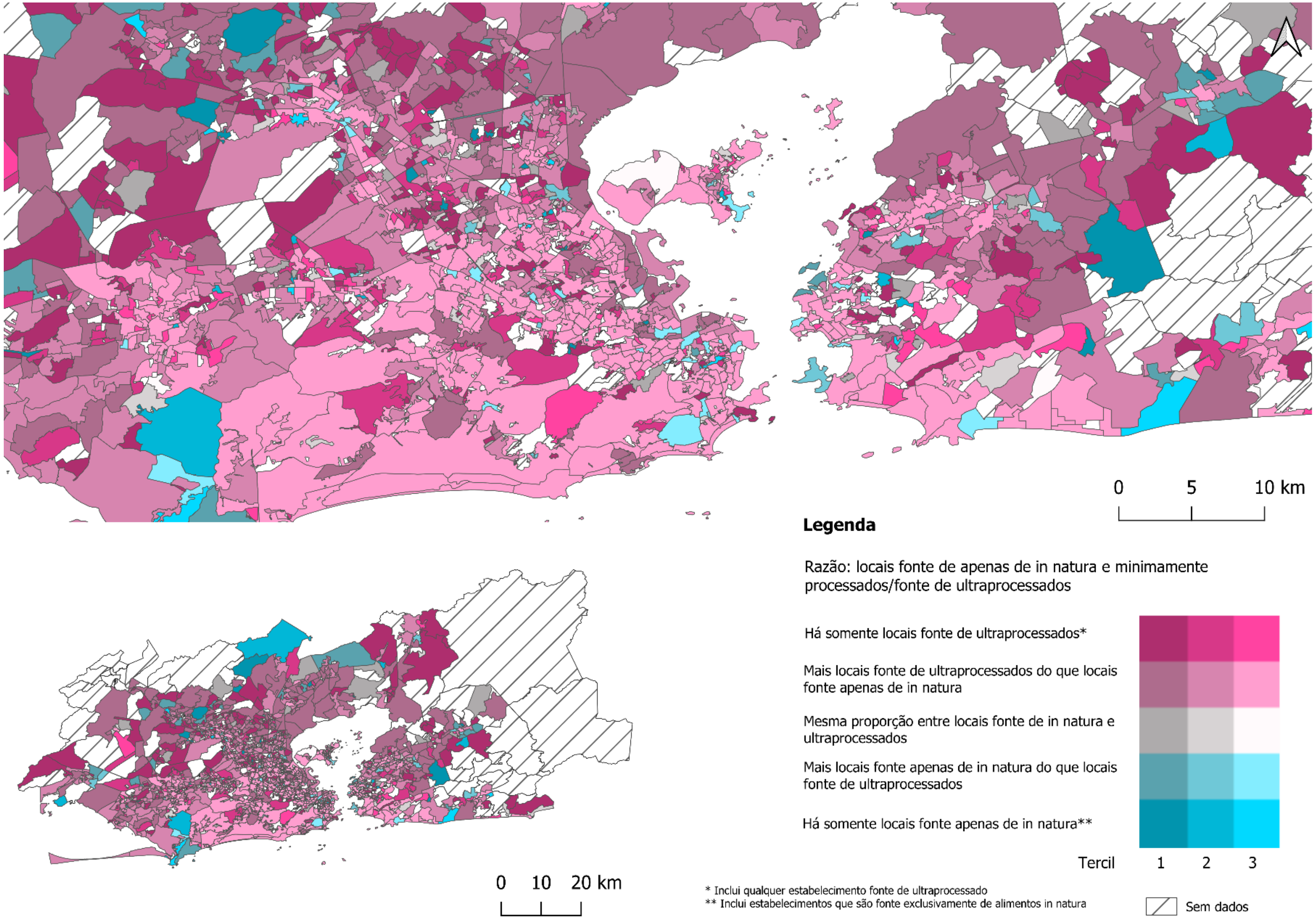

Figura Suplementar 17. Distribuição espacial do indicador de saudabilidade do ambiente alimentar (razão entre locais fonte de apenas de in natura e minimamente processados e fonte de ultraprocessados) na Região Metropolitana de Salvador.

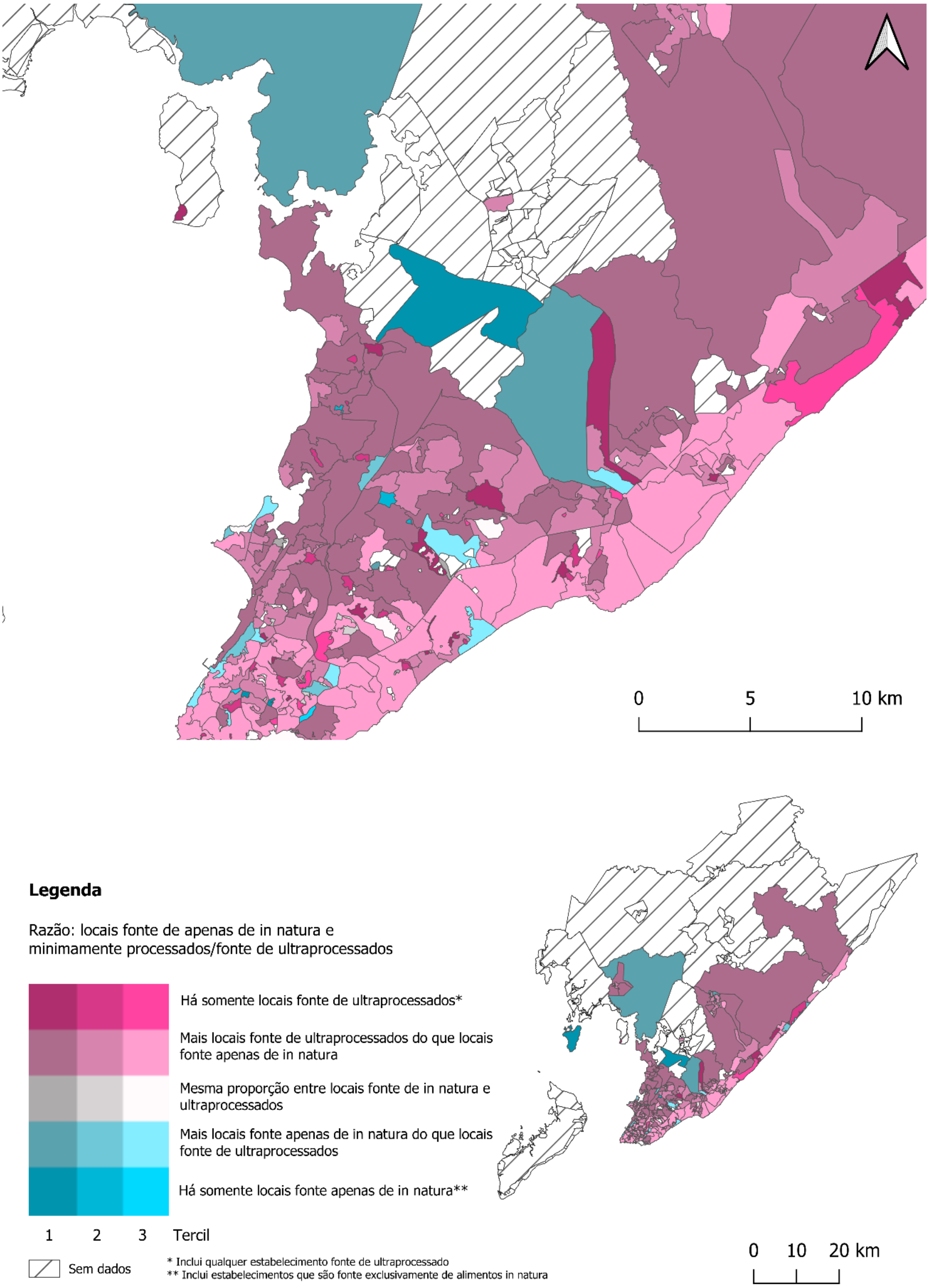

Figura Suplementar 18. Distribuição espacial do indicador de saudabilidade do ambiente alimentar (razão entre locais fonte de apenas de in natura e minimamente processados e fonte de ultraprocessados) na Região Metropolitana de São Luis.

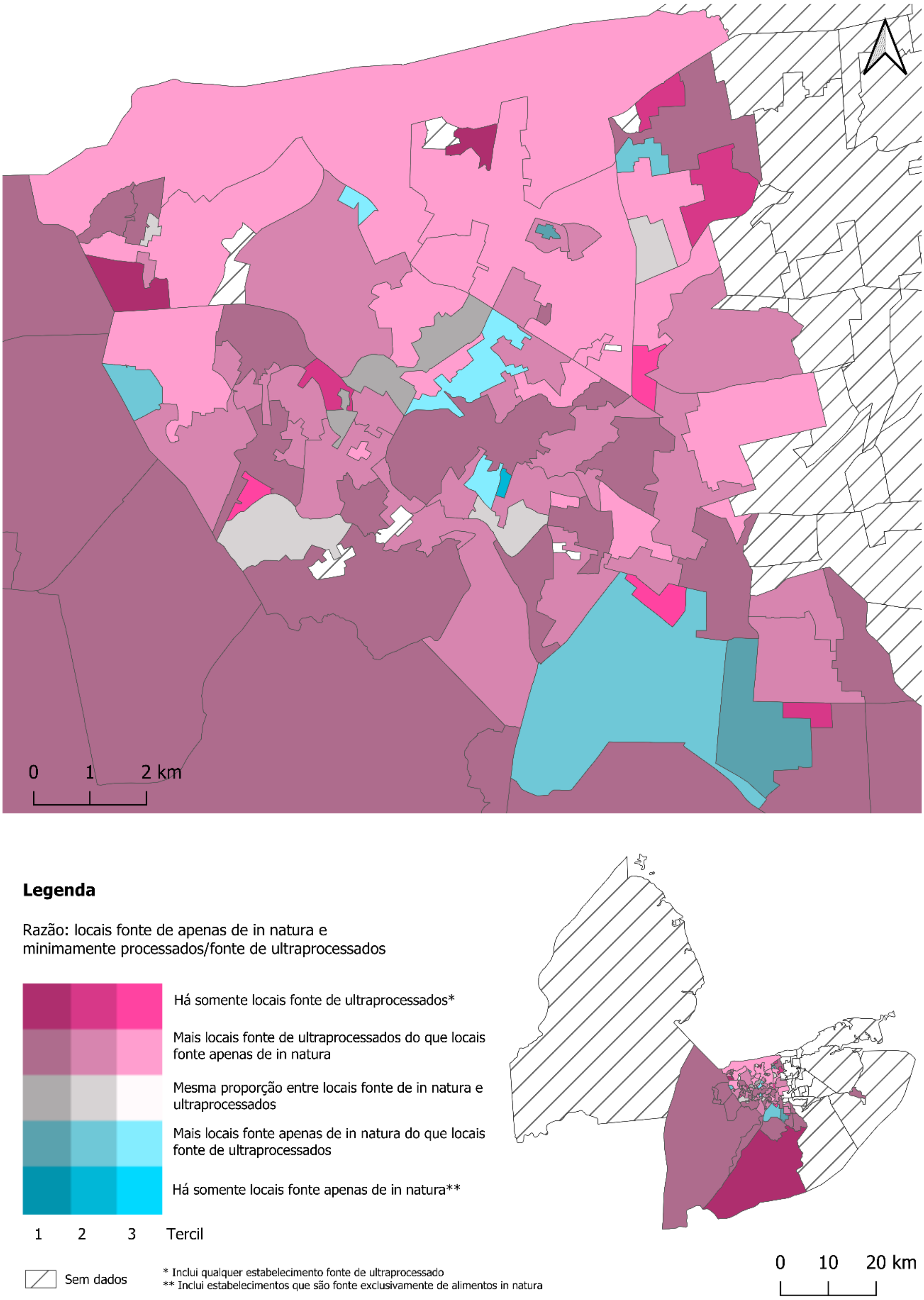

Figura Suplementar 19. Distribuição espacial do indicador de saudabilidade do ambiente alimentar (razão entre locais fonte de apenas de in natura e minimamente processados e fonte de ultraprocessados) na Região Metropolitana de Sorocaba.

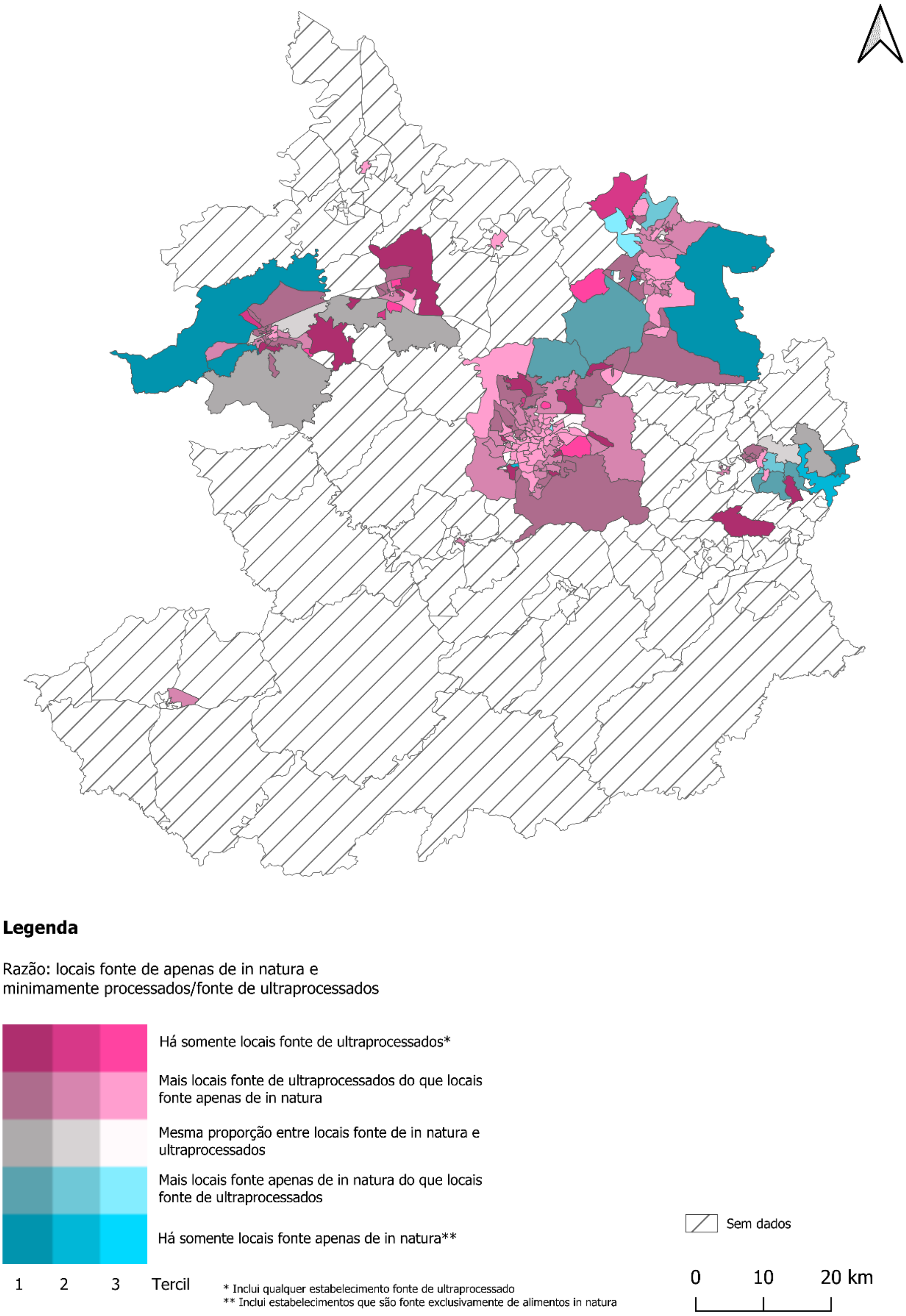

Figura Suplementar 20. Distribuição espacial do indicador de saudabilidade do ambiente alimentar (razão entre locais fonte de apenas de in natura e minimamente processados e fonte de ultraprocessados) na Região Metropolitana de Teresina e Timon.

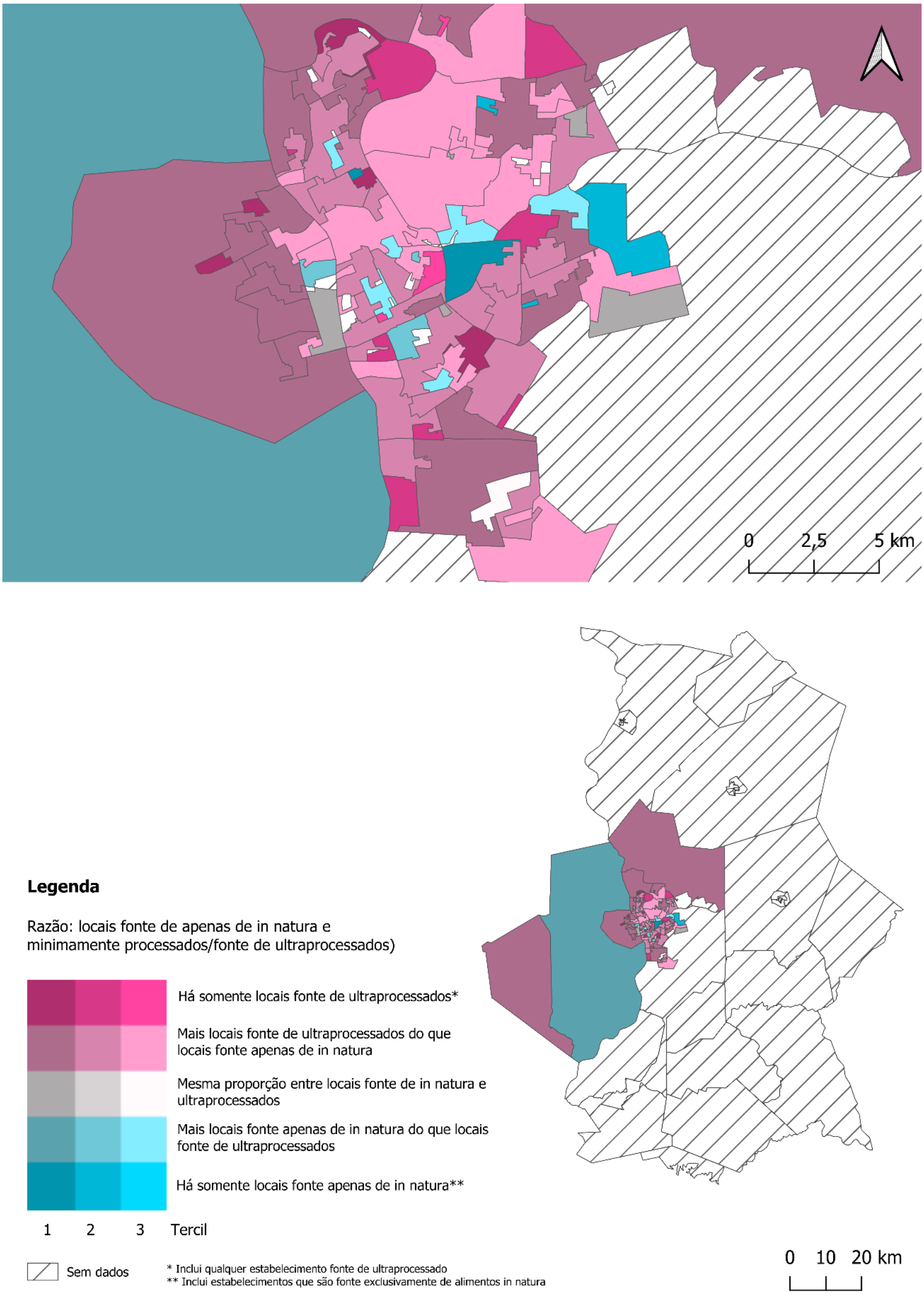

Figura Suplementar 21. Distribuição espacial do indicador de saudabilidade do ambiente alimentar (razão entre locais fonte de apenas de in natura e minimamente processados e fonte de ultraprocessados) na Região Metropolitana do Vale do Paraíba e Litoral Norte.

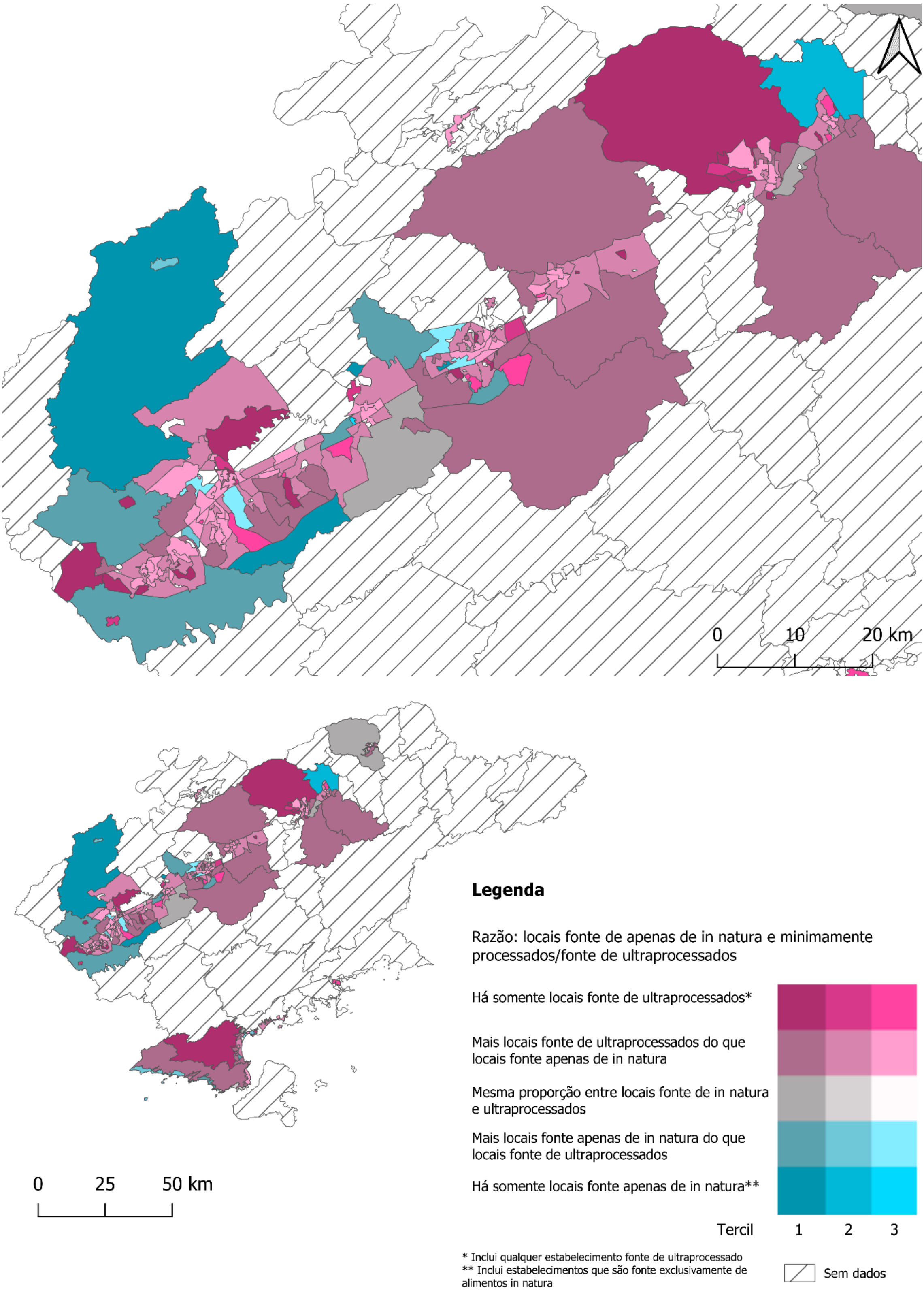

Figura Suplementar 22. Distribuição espacial do indicador de saudabilidade do ambiente alimentar (razão entre locais fonte de apenas de in natura e minimamente processados e fonte de ultraprocessados) na Região Metropolitana do Vale do Rio Cuiabá.

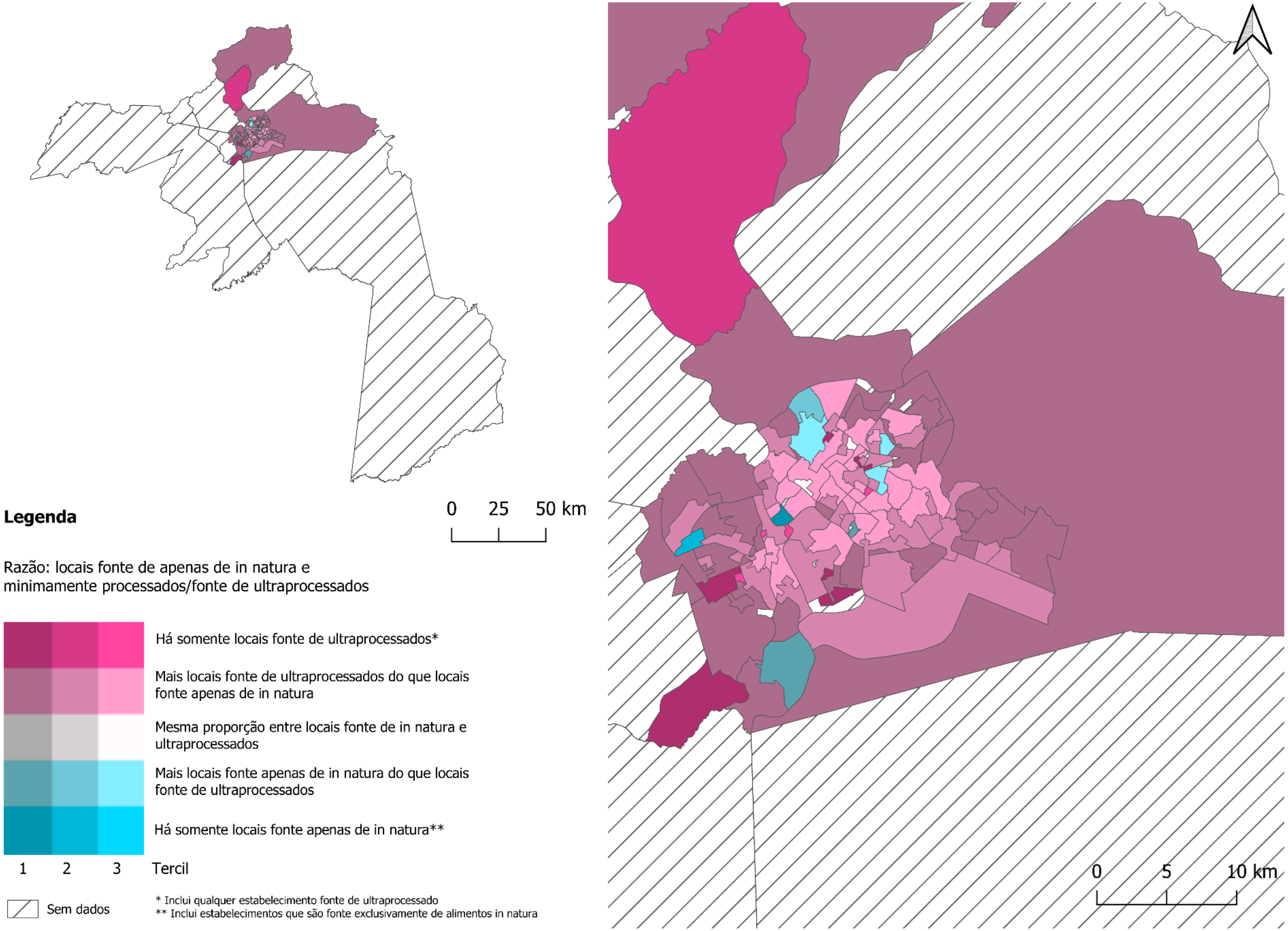

Figura Suplementar 23. Distribuição espacial do indicador de saudabilidade do ambiente alimentar (razão entre locais fonte de apenas de in natura e minimamente processados e fonte de ultraprocessados) na Região Metropolitana de Vitória.

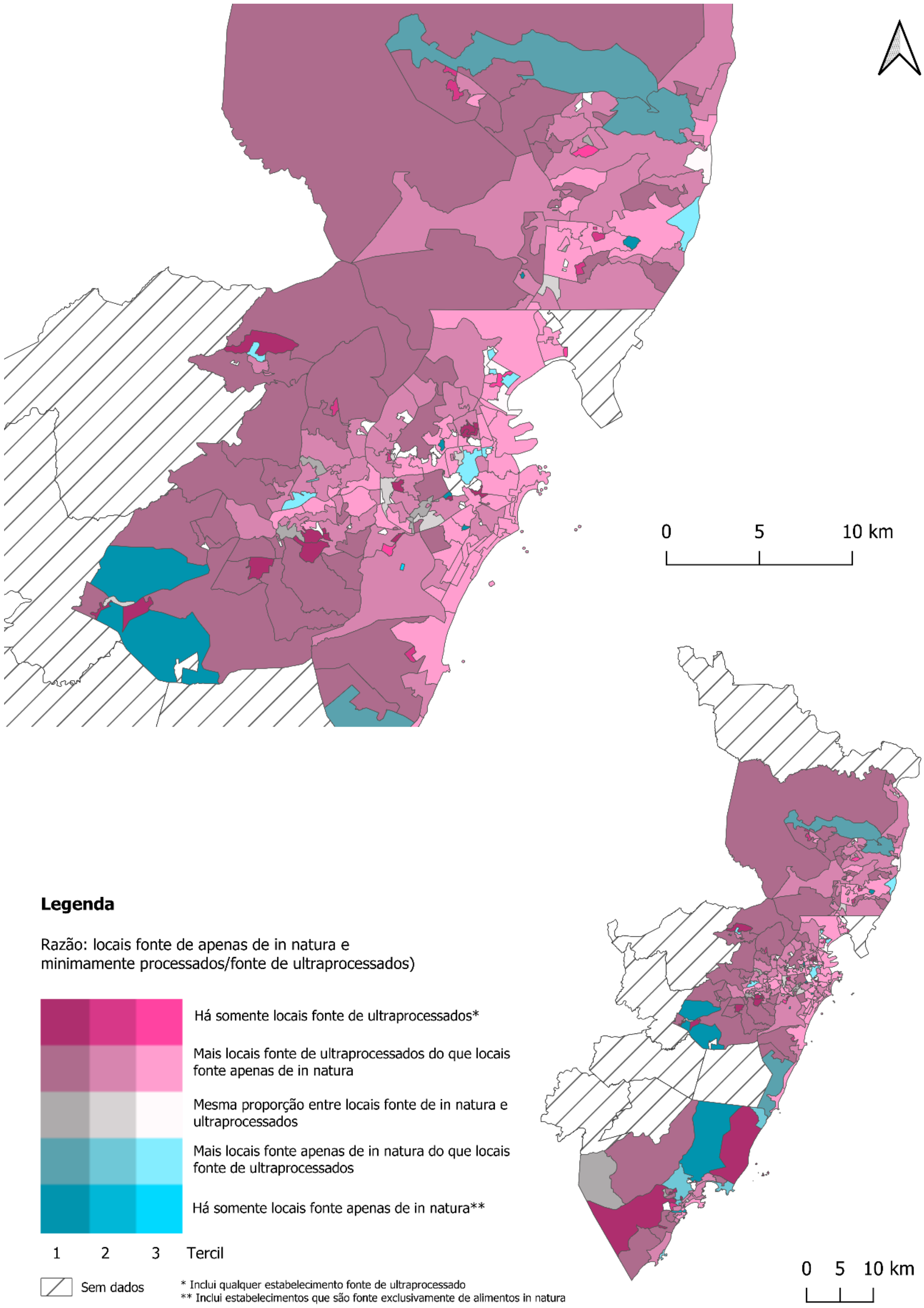

Supplement: Supplementary file 2 [file 1980-5497-rbepid-29-e260040-Suppl02-pt.pdf]
