# Supplementary material for: Territorial inequalities in the food environment of Brazilian metropolitan regions
Source: Rev Bras Epidemiol. 2026 Aug 17;29:e260040. doi: 10.1590/1980-549720260040 (PMC13480622; doi:10.1590/1980-549720260040)
Supplement: Supplementary file 3 [file 1980-5497-rbepid-29-e260040-Suppl01.docx]

**Supplementary Table 1.** Number of Human Development Units and average HDI in the Metropolitan Regions and Integrated Economic Development Regions studied

| Metropolitan Region / Integrated Economic Development Region | UDH Number | Average HDI (95%IC) |
| --- | --- | --- |
| Baixada Santista (SP) | 203 | 0.77 (0.76; 0.78) |
| Belém (PA) | 192 | 0.76 (0.74; 0.77) |
| Belo Horizonte (MG) | 439 | 0.75 (0.74; 0.76) |
| Campinas (SP) | 364 | 0.79 (0.78; 0.80) |
| Curitiba (PR) | 251 | 0.76 (0.75; 0.77) |
| Distrito Federal and surrounding (DF and MG) | 290 | 0.78 (0.77; 0.79) |
| Florianópolis (SC) | 223 | 0.81 (0.80; 0.82) |
| Fortaleza (CE) | 280 | 0.73 (0.72; 0.74) |
| Goiânia (GO) | 223 | 0.79 (0.77; 0.80) |
| Maceió (AL) | 111 | 0.74 (0.72; 0.76) |
| Manaus (AM) | 203 | 0.75 (0.73; 0.76) |
| Natal (RN) | 94 | 0.75 (0.73; 0.77) |
| Petrolina-Juazeiro (BA and PE) | 64 | 0.70 (0.68; 0.73) |
| Porto Alegre (RS) | 516 | 0.75 (0.74; 0.76) |
| Recife (PE) | 373 | 0.73 (0.72; 0.74) |
| Rio de Janeiro (RJ) | 1643 | 0.75 (0.75; 0.76) |
| Salvador (BA) | 271 | 0.76 (0.75; 0.77) |
| São Luís (MA) | 90 | 0.78 (0.77; 0.80) |
| São Paulo (SP) | 2202 | 0.77 (0.77; 0.77) |
| Sorocaba (SP) | 203 | 0.77 (0.77; 0.78) |
| Teresina and Timon (PI and MA) | 112 | 0.72 (0.70; 0.73) |
| Vale do Paraíba and Litoral Norte (SP) | 254 | 0.78 (0.77; 0.79) |
| Vale do Rio Cuiabá (MT) | 123 | 0.78 (0.76; 0.79) |
| Vitória (ES) | 253 | 0.76 (0.75; 0.77) |

**Supplementary Table 2.** Losses resulting from the georeferencing of the 2023 Annual Social Information Report, according to Metropolitan Region or Integrated Economic Development Region.

| **Annual List of Social Information 2023** | | | |
| --- | --- | --- | --- |
| Metropolitan Region and Integrated Economic Development Region | Number of food establishments | | % losses |
|  | Before georeferencing | After georeferencing |  |
| Baixada Santista (SP) | 8836 | 8789 | 0.5 |
| Belém (PA) | 5182 | 5129 | 1.0 |
| Belo Horizonte (MG) | 23545 | 23035 | 2.2 |
| Distrito Federal and surrounding (DF and MG) | 22584 | 22008 | 2.6 |
| Campinas (SP) | 15969 | 15837 | 0.8 |
| Curitiba (PR) | 20772 | 20644 | 0.6 |
| Florianópolis (SC) | 9225 | 9180 | 0.5 |
| Fortaleza (CE) | 13986 | 13863 | 0.9 |
| Goiânia (GO) | 12447 | 12219 | 1.8 |
| Maceió (AL) | 4417 | 4402 | 0.3 |
| Manaus (AM) | 7090 | 7072 | 0.3 |
| Natal (RN) | 5417 | 5353 | 1.2 |
| Petrolina-Juazeiro (BA and PE) | 2257 | 2223 | 1.5 |
| Porto Alegre (RS) | 17119 | 16891 | 1.3 |
| Recife (PE) | 12536 | 12354 | 1.5 |
| Rio de Janeiro (RJ) | 40036 | 39622 | 1.0 |
| Salvador (BA) | 12770 | 12692 | 0.6 |
| São Paulo (SP) | 107618 | 106725 | 0.8 |
| São Luís (MA) | 4481 | 4409 | 1.6 |
| Sorocaba (SP) | 7918 | 7837 | 1.0 |
| Teresina and Timon (PI and MA) | 4551 | 4548 | 0.1 |
| Vale do Paraíba and Litoral Norte (SP) | 11220 | 11095 | 1.1 |
| Vale do Rio Cuiabá (MT) | 4990 | 4881 | 2.2 |
| Vitória (ES) | 8820 | 8689 | 1.5 |
| Total | 388075 | 383786 | 1.1 |

Supplmentary Box 1. Food purchasing places as per the recommendations of the Dietary Guidelines for the Brazilian population, Brazil and Brazilian Regions.

| Indicators | Brazil | North | Northeast | Southeast | South | Midwest |
| --- | --- | --- | --- | --- | --- | --- |
| Places source of unprocessed or minimally processed foods and processed culinary ingredients | Supermarkets  Produce  markets/greengrocers  Butcher shops Food street vendors Restaurants  Fish markets | Supermarkets  Produce  markets/greengrocers  Butcher shops Food street vendors Restaurants  Fish markets | Supermarkets  Produce  markets/greengrocers  Butcher shops Food street vendors Restaurants  Fish markets | Supermarkets  Produce  markets/greengrocers  Butcher shops Food street vendors Restaurants  Fish markets | Supermarkets  Produce  markets/greengrocers  Butcher shops Food street vendors Restaurants  Fish markets  Canteens | Supermarkets  Produce  markets/greengrocers  Butcher shops Food street vendors Restaurants  Fish markets |
| Places source of only unprocessed or minimally processed foods and processed culinary ingredients | Produce  markets/greengrocers  Butcher shops Food street vendors Restaurants  Fish markets | Produce  markets/greengrocers  Butcher shops Food street vendors Restaurants  Fish markets | Produce  markets/greengrocers  Butcher shops Food street vendors Restaurants  Fish markets | Produce  markets/greengrocers  Butcher shops Restaurants  Fish markets | Produce  markets/greengrocers  Butcher shops Food street vendors Restaurants  Fish markets  Canteens | Produce  markets/greengrocers  Butcher shops Restaurants  Fish markets |
| Places source of processed foods | Bakeries  Bars  Convenience stores  Dairy retailers  Canteens | Bakeries  Bars  Convenience stores  Dairy retailers | Bakeries  Food street vendors  Dairy retailers  Canteens | Bakeries  Food street vendors  Bars  Convenience stores  Dairy retailers  Canteens | Bakeries  Dairy retailers | Bakeries  Food street vendors  Convenience stores  Dairy retailers |
| Places source of ultra-processed foods | Supermarkets  Minimarkets  Bakeries  Snack bars  Bars  Convenience stores  Candy stores  Canteens  Frozen and ready-to-eat food vendors  Others | Supermarkets  Minimarkets  Bakeries  Snack bars  Bars  Convenience stores  Candy stores  Dairy retailers  Canteens  Frozen and ready-to-eat food vendors  Others | Minimarkets  Snack bars  Bars  Convenience stores  Candy stores  Canteens  Frozen and ready-to-eat food vendors  Others | Supermarkets  Minimarkets  Snack bars  Bars  Convenience stores  Candy stores  Canteens  Frozen and ready-to-eat food vendors  Others | Supermarkets  Minimarkets  Bakeries  Snack bars  Bars  Convenience stores  Candy stores  Canteens  Frozen and ready-to-eat food vendors  Others | Supermarkets  Minimarkets  Bakeries  Snack bars  Bars  Convenience stores  Candy stores  Dairy retailers  Canteens  Frozen and ready-to-eat food vendors  Others |
| Places source of only ultra-processed foods | Minimarkets  Snack bars  Candy stores  Frozen and ready-to-eat food vendors  Others | Minimarkets  Snack bars  Convenience stores  Candy stores  Canteens  Frozen and ready-to-eat food vendors  Others | Minimarkets  Snack bars  Bars  Convenience stores  Candy stores  Frozen and ready-to-eat food vendors  Others | Minimarkets  Snack bars  Candy stores  Frozen and ready-to-eat food vendors  Others | Minimarkets  Snack bars  Bars  Candy stores  Frozen and ready-to-eat food vendors  Others | Minimarkets  Snack bars  Bars  Candy stores  Canteens  Frozen and ready-to-eat food vendors  Others |
