# Supplementary material for: Territorial inequalities in the food environment of Brazilian metropolitan regions
Source: Rev Bras Epidemiol. 2026 Aug 17;29:e260040. doi: 10.1590/1980-549720260040 (PMC13480622; doi:10.1590/1980-549720260040)
Supplement: Supplementary file 4 [file 1980-5497-rbepid-29-e260040-Suppl02.docx]

Supplementary Figure 1. Spatial distribution of the food environment healthiness indicator (ratio of outlets offering only unprocessed and minimally processed foods to outlets offering ultra-processed foods) in the Baixada Santista Metropolitan Region.

Supplementary Figure 2. Spatial distribution of the food environment healthiness indicator (ratio of outlets offering only unprocessed and minimally processed foods to outlets offering ultra-processed foods) in the Belém Metropolitan Region.

Supplementary Figure 3. Spatial distribution of the food environment healthiness indicator (ratio of outlets offering only unprocessed and minimally processed foods to outlets offering ultra-processed foods) in the Belo Horizonte Metropolitan Region.

Supplementary Figure 4. Spatial distribution of the food environment healthiness indicator (ratio of outlets offering only unprocessed and minimally processed foods to outlets offering ultra-processed foods) in the Campinas Metropolitan Region.

Supplementary Figure 5. Spatial distribution of the food environment healthiness indicator (ratio of outlets offering only unprocessed and minimally processed foods to outlets offering ultra-processed foods) in the Curitiba Metropolitan Region.

Supplementary Figure 6. Spatial distribution of the food environment healthiness indicator (ratio of outlets offering only unprocessed and minimally processed foods to outlets offering ultra-processed foods) in the Integrated Economic Development Region of Distrito Federal and surrounding.

Supplementary Figure 7. Spatial distribution of the food environment healthiness indicator (ratio of outlets offering only unprocessed and minimally processed foods to outlets offering ultra-processed foods) in the Florianópolis Metropolitan Region.

Supplementary Figure 8. Spatial distribution of the food environment healthiness indicator (ratio of outlets offering only unprocessed and minimally processed foods to outlets offering ultra-processed foods) in the Fortaleza Metropolitan Region.

Supplementary Figure 9. Spatial distribution of the food environment healthiness indicator (ratio of outlets offering only unprocessed and minimally processed foods to outlets offering ultra-processed foods) in the Goiânia Metropolitan Region.

Supplementary Figure 10. Spatial distribution of the food environment healthiness indicator (ratio of outlets offering only unprocessed and minimally processed foods to outlets offering ultra-processed foods) in the Maceió Metropolitan Region.

Supplementary Figure 11. Spatial distribution of the food environment healthiness indicator (ratio of outlets offering only unprocessed and minimally processed foods to outlets offering ultra-processed foods) in the Manaus Metropolitan Region.

Supplementary Figure 12. Spatial distribution of the food environment healthiness indicator (ratio of outlets offering only unprocessed and minimally processed foods to outlets offering ultra-processed foods) in the Natal Metropolitan Region.

Supplementary Figure 13. Spatial distribution of the food environment healthiness indicator (ratio of outlets offering only unprocessed and minimally processed foods to outlets offering ultra-processed foods) in the Integrated Economic Development Region of Petrolina-Juazeiro.

Supplementary Figure 14. Spatial distribution of the food environment healthiness indicator (ratio of outlets offering only unprocessed and minimally processed foods to outlets offering ultra-processed foods) in the Porto Alegre Metropolitan Region.

Supplementary Figure 15. Spatial distribution of the food environment healthiness indicator (ratio of outlets offering only unprocessed and minimally processed foods to outlets offering ultra-processed foods) in the Recife Metropolitan Region.

Supplementary Figure 16. Spatial distribution of the food environment healthiness indicator (ratio of outlets offering only unprocessed and minimally processed foods to outlets offering ultra-processed foods) in the Rio de Janeiro Metropolitan Region.

Supplementary Figure 17. Spatial distribution of the food environment healthiness indicator (ratio of outlets offering only unprocessed and minimally processed foods to outlets offering ultra-processed foods) in the Salvador Metropolitan Region.

Supplementary Figure 18. Spatial distribution of the food environment healthiness indicator (ratio of outlets offering only unprocessed and minimally processed foods to outlets offering ultra-processed foods) in the São Luis Metropolitan Region.

Supplementary Figure 19. Spatial distribution of the food environment healthiness indicator (ratio of outlets offering only unprocessed and minimally processed foods to outlets offering ultra-processed foods) in the Sorocaba Metropolitan Region.

Supplementary Figure 20. Spatial distribution of the food environment healthiness indicator (ratio of outlets offering only unprocessed and minimally processed foods to outlets offering ultra-processed foods) in the Integrated Economic Development Region of Teresina and Timon.

Supplementary Figure 19. Spatial distribution of the food environment healthiness indicator (ratio of outlets offering only unprocessed and minimally processed foods to outlets offering ultra-processed foods) in the Vale do Paraíba and Litoral Norte Metropolitan Region.

Supplementary Figure 22. Spatial distribution of the food environment healthiness indicator (ratio of outlets offering only unprocessed and minimally processed foods to outlets offering ultra-processed foods) in the Vale do Rio Cuiabá Metropolitan Region.

Supplementary Figure 23. Spatial distribution of the food environment healthiness indicator (ratio of outlets offering only unprocessed and minimally processed foods to outlets offering ultra-processed foods) in the Vitória Metropolitan Region.
