# Supplementary material for: Territorial inequalities in the food environment of Brazilian metropolitan regions
Source: Rev Bras Epidemiol. 2026 Aug 17;29:e260040. doi: 10.1590/1980-549720260040 (PMC13480622; doi:10.1590/1980-549720260040)
Supplement: Translation

# Translation: Desigualdades territoriais do ambiente alimentar de regiões metropolitanas brasileiras

**DOI:** 10.1590/1980-549720260040.2

**Authors:** Laura Luciano Scaciota, Claudio Makoto Kanai, Marcos Anderson Lucas da Silva, Renata Bertazzi Levy

**Affiliations:** I Universidade de São Paulo, Núcleo de Pesquisas Epidemiológicas em Nutrição e Saúde – São Paulo (SP), Brasil.; II Universidade de São Paulo, Faculdade de Medicina, Departamento de Medicina Preventiva – São Paulo (SP), Brasil; III Universidade de São Paulo, Faculdade de Saúde Pública, Departamento de Nutrição – São Paulo (SP), Brasil.; IV University of Salamanca, Institute of Biomedical Research of Salamanca – Salamanca, Espanha.

**Keywords:** Ambiente alimentar, Disparidades socioeconômicas em saúde, Abastecimento de alimentos, Acesso a alimentos saudáveis

## Abstract

**Objetivo:**

Descrever o ambiente alimentar varejista das regiões metropolitanas brasileiras com indicadores socioeconômicos intramunicipais, utilizando a classificação Locais-Nova.

**Métodos:**

Foram utilizados dados da Relação Anual de Informações Sociais de 2023, tendo como unidade de análise as Unidades de Desenvolvimento Humano (UDH). Os estabelecimentos de alimentos foram georreferenciados e associados às UDH. Aplicou-se a metodologia Locais-Nova, que classifica os locais de aquisição de alimentos de acordo com o grau de processamento e propõe cinco indicadores do ambiente alimentar. Calculou-se o percentual de cada indicador para as UDH, estratificado em tercis do Índice de Desenvolvimento Humano Municipal (IDHM), segundo a distribuição das UDH, as macrorregiões e entre capitais e não capitais. Foi criado um indicador de saudabilidade do ambiente alimentar que mede a competição entre locais saudáveis e não saudáveis, utilizado para descrição espacial por meio de mapas.

**Resultados:**

Foram avaliadas 8.977 UDH em 24 regiões metropolitanas, com IDHM médio de 0,76 e média de 42,8 estabelecimentos de alimentos por UDH. A maioria dos locais de aquisição foi classificada como fonte de ultraprocessados (64,9%), com maior proporção no primeiro tercil de IDHM (67,5%) em relação ao 3º (63,1%). Observou-se menor presença de locais fonte de in natura no primeiro tercil de IDHM comparado ao 3º (25,8 vs. 32,3%). O Sudeste apresentou maior percentual de locais fonte de processados (17,3%). As não capitais concentraram maior proporção de locais fonte de processados em relação às capitais.

**Conclusão:**

Constatou-se elevada prevalência de locais fonte de alimentos ultraprocessados, sobretudo em territórios com piores situações socioeconômicas.

## INTRODUÇÃO

O acesso aos alimentos é influenciado pelo território em que as pessoas vivem, tanto pela presença e concentração de locais de aquisição quanto pelo perfil dos alimentos comercializados^1,2^. A quantidade e o tipo de estabelecimentos tendem a refletir as condições socioeconômicas do ambiente em que se inserem, revelando padrões de desigualdade na distribuição territorial da oferta de alimentos^3^.

Ambientes com maior densidade de estabelecimentos que comercializam predominantemente ultraprocessados estão associados a maior compra e consumo desses alimentos^4,5^. As características intrínsecas dos ultraprocessados, somadas às suas práticas comerciais, geram efeitos nocivos à saúde dos consumidores^6^ e contribuem para sua ampla presença nos ambientes alimentares^7^.

Identificar os territórios mais vulneráveis socioeconomicamente e analisar a distribuição de locais de aquisição de alimentos nesses espaços é essencial para subsidiar o planejamento urbano e a elaboração de políticas públicas voltadas aos determinantes sociais e comerciais da saúde.

Enquanto estudos internacionais analisam o ambiente alimentar em escala nacional^8,9^, a produção científica brasileira permanece concentrada em recortes geográficos restritos e focalizados na região Sudeste^10-13^. Além disso, estudos sobre ambientes alimentares que utilizam dados secundários adotam ampla variedade de metodologias para identificar quais locais de aquisição de alimentos são ou não considerados saudáveis^14-17^. Essa heterogeneidade persiste mesmo entre estudos baseados na classificação Nova^18-21^.

Recentemente, uma metodologia inovadora foi proposta para a categorização dos locais de aquisição de alimentos. Denominada Locais-Nova, ela incorpora as recomendações do Guia Alimentar para a População Brasileira e é aplicável a todas as regiões do Brasil, permitindo comparações entre diferentes contextos^22^. O objetivo deste estudo foi descrever o ambiente alimentar varejista das regiões metropolitanas brasileiras segundo indicadores socioeconômicos intramunicipais, de acordo com a classificação Locais-Nova.

## MÉTODOS

### Fonte de dados

Este é um estudo ecológico descritivo realizado em todas as Regiões Metropolitanas (RM) e Regiões Integradas de Desenvolvimento Econômico (RIDE) brasileiras com indicadores socioeconômicos intramunicipais. Foram utilizados dados do Atlas do Desenvolvimento Humano nas Regiões Metropolitanas Brasileiras, da Relação Anual de Informações Sociais (RAIS) de 2023^23^ e dos Correios^24^.

#### Atlas do desenvolvimento humano nas regiões metropolitanas brasileiras

O Atlas do Desenvolvimento Humano é uma plataforma elaborada pelo Instituto de Pesquisas Econômicas Aplicadas (Ipea), pelo Programa das Nações Unidas para o Desenvolvimento (Pnud) e pela Fundação João Pinheiro^25^. O Atlas produziu as Unidades de Desenvolvimento Humano (UDH), divisões espaciais formadas pelo agrupamento de setores censitários com características socioeconômicas semelhantes, garantindo maior homogeneidade socioeconômica do território. Esse agrupamento permite avaliar desigualdades dentro do mesmo município. As UDH representam as menores territorialidades brasileiras com Índice de Desenvolvimento Humano Municipal (IDHM) calculado e foram a unidade de análise deste estudo^26^.

O IDHM é baseado no Índice de Desenvolvimento Humano (IDH) global e adaptado à realidade brasileira. O índice é composto pela média geométrica de três subíndices: longevidade (expectativa de vida ao nascer), educação (nível educacional da população adulta e fluxo escolar da população jovem) e renda (renda per capita). O IDHM varia de 0 a 1, e quanto mais próximo de 1, melhor é o indicador de desenvolvimento humano da UDH^27^.

O Atlas disponibiliza a malha das UDH (formato *shapefile*) para 24 RM e RIDE brasileiras (Tabela Suplementar 1).

#### Relação Anual de Informações Sociais

A RAIS é um relatório de informações socioeconômicas solicitado pelo Ministério do Trabalho e Emprego aos empregadores e pessoas jurídicas^23^. Os proprietários dos estabelecimentos preenchem anualmente uma declaração com informações como nome do estabelecimento, número de empregados, unidade da federação, município, Código de Endereçamento Postal (CEP) e Classificação Nacional de Atividades Econômicas (CNAE)^28^.

A CNAE é o instrumento de padronização nacional dos códigos de atividade econômica para estabelecimentos comerciais, permitindo identificar diferentes tipos de comércios.

#### Correios

O Diretório Nacional de Endereços é uma base de dados dos Correios que contém os endereços completos do Brasil, incluindo informações como: estado, cidade, bairro, nome da rua, CEP e coordenadas geográficas. A base foi adquirida em 2021 no formato .csv^24^.

### Tratamento e análise dos dados

O estudo foi realizado em oito etapas. Na primeira, foram selecionados os estabelecimentos comerciais de interesse na base da RAIS. Foram incluídos CNAEs de: supermercados (incluindo os hipermercados), minimercados, padarias (fabricação e revenda), varejistas de frios e laticínios, bombonieres, açougues, peixarias, hortifrutis, lojas de conveniência, venda de alimentos congelados (delicatessen e delivery), restaurantes, lanchonetes e bares (com e sem entretenimento). Não foram considerados CNAEs de cantinas, por serem espaços privados acessados apenas por frequentadores de instituições como escolas, hospitais e locais de trabalho; de ambulantes de alimentos, por serem mal representados pela RAIS devido à informalidade; e de comércios de bebidas, como depósitos e distribuidoras, por não serem classificados segundo a Locais-Nova.

A segunda etapa consistiu na agregação das coordenadas geográficas dos Correios aos estabelecimentos comerciais, por meio do CEP.

Na terceira etapa, os estabelecimentos foram georreferenciados no software QGIS, utilizando as coordenadas obtidas na etapa anterior, criando uma camada vetorial de pontos. O percentual médio de perdas decorrentes do georreferenciamento, como coordenadas inválidas, foi de 1,1% (Tabela Suplementar 2).

A quarta etapa foi a associação das informações da UDH aos estabelecimentos comerciais pelo recurso "associação de dados por localização", o cálculo do número de cada tipo de estabelecimento em cada UDH, e o cálculo da área das UDH em km^2^.

Na quinta etapa, os estabelecimentos foram agrupados segundo indicadores propostos pela metodologia Locais-Nova, baseada nas recomendações do Guia Alimentar para a População Brasileira.

O sistema criou 5 indicadores:

1. Locais de aquisição fonte de alimentos que devem ser a base da alimentação (fonte de aquisição de alimentos in natura ou minimamente processados e de ingredientes culinários, doravante denominado locais fonte de in natura);
2. Locais de aquisição identificados apenas como fonte de alimentos que devem ser a base da alimentação (fonte de aquisição apenas de alimentos in natura ou minimamente processados e ingredientes culinários, doravante denominado locais fonte apenas de in natura);
3. Locais de aquisição fonte de alimentos que devem ser limitados (fonte de aquisição de alimentos processados, doravante denominado locais fonte de processados);
4. Locais de aquisição fonte de alimentos que devem ser evitados (fonte de aquisição de alimentos ultraprocessados, doravante denominado locais fonte de ultraprocessados); e
5. Locais de aquisição identificados apenas como fonte de alimentos que devem ser evitados (fontes de aquisição apenas de alimentos ultraprocessados, doravante denominado locais fonte apenas de ultraprocessados).

A fim de captar diferenças regionais, a Locais-Nova fornece indicadores específicos para cada macrorregião, adotados neste estudo (Quadro Suplementar 1). Detalhes sobre a criação dos indicadores podem ser encontrados em Silva et al.^22^.

A sexta etapa foi a análise descritiva dos dados. Foram calculados os percentuais de cada indicador nas UDH e realizada a estratificação por tercis de IDHM (quanto maior o tercil, maior o IDHM), considerando a distribuição total das UDH, por macrorregião e entre capitais e não capitais. As análises foram realizadas no software *Stata*/SE 16.1^27^.

A sétima etapa consistiu na criação do indicador de saudabilidade do ambiente alimentar, definido como a razão entre o número de "Locais fonte apenas de in natura" (numerador) e o número de "Locais fonte de ultraprocessados" (denominador), conforme a classificação Locais-Nova. Como um mesmo estabelecimento pode ser simultaneamente fonte de alimentos in natura e de ultraprocessados, o numerador inclui exclusivamente locais que não comercializam ultraprocessados, enquanto o denominador inclui todos os locais fonte desses produtos.

O indicador de saudabilidade baseou-se na recomendação de evitar o consumo de ultraprocessados e no entendimento de que sua oferta representa um obstáculo para a alimentação adequada e saudável^29^. Valores mais elevados indicam maior predominância relativa de locais que ofertam exclusivamente alimentos in natura em relação àqueles que ofertam ultraprocessados, caracterizando ambientes mais favoráveis à alimentação saudável.

Por fim, na oitava etapa, foi descrita a distribuição espacial dos dados nas UDH das 24 RM e RIDE. Foram elaborados mapas bivariados com base no indicador de saudabilidade do ambiente alimentar (razão entre locais fonte apenas de in natura e locais fonte de ultraprocessados) e nos tercis de IDHM, calculados separadamente em cada região metropolitana, considerando a distribuição das UDH em cada uma delas.

O indicador de saudabilidade do ambiente alimentar divide-se em cinco categorias, cada uma com uma cor correspondente:

1. UDH com locais fonte de ultraprocessados, sem a presença de locais fonte apenas de in natura (rosa escuro);
2. UDH com predominância de locais fonte de ultraprocessados em relação aos locais fonte apenas de in natura (rosa claro);
3. UDH com a mesma proporção entre locais fonte de ultraprocessados e locais fonte apenas de in natura (branco);
4. UDH com predominância de locais fonte apenas de in natura em relação aos locais fonte de ultraprocessados (azul claro);
5. UDH com locais fonte apenas de in natura, sem a presença de locais fonte de ultraprocessados (azul escuro).

Quanto maior o IDHM, mais brilhante é a cor utilizada.

As análises espaciais foram realizadas no QGIS versão 3.22.14, utilizando o Sistema de Referências de Coordenadas (SRC) SIRGAS 2000. Para cálculo da área das UDH foi utilizada a Projeção de Albers^30^.

#### Declaração de disponibilidade de dados:

Os dados que dão suporte aos resultados deste estudo estão disponíveis publicamente e podem ser acessados pelo portal do Atlas Brasil e do Ministério do Trabalho. O conjunto de dados não está publicamente disponível devido à integração de bancos de dados realizados pelos autores.

## RESULTADOS

Foram avaliadas 8.977 UDH nas 24 regiões metropolitanas, com IDHM médio de 0,76. As UDH de menor IDHM apresentam maior população média (8.811,3) e área territorial mais extensa (25,8 km^2^), enquanto as de maior IDHM apresentam menor população (7.446,6) e área menor (2,4 km^2^), mas maior número médio de estabelecimentos de alimentos (75,7).

A maior parte dos locais de aquisição é fonte de ultraprocessados (64,9%). Há diferença de aproximadamente 5% entre o 1º (67,5 e 58,6%) e o 3º (63,1 e 53,9%) tercil de IDHM para locais fonte de ultraprocessados e fonte apenas de ultraprocessados, respectivamente. A presença de estabelecimentos fonte apenas de in natura é 34% mais elevada nas UDH de maior IDHM (29,6%) em relação às de menor IDHM (22,1%) (Tabela 1).

**Tabela 1:** Características das Unidades de Desenvolvimento Humano e percentual de estabelecimentos de acordo com categorias Locais-Nova, por tercis de IDHM, nas regiões metropolitanas com indicadores socioeconômicos intramunicipais: 2023.

		Nível do Índice de Desenvolvimento Humano Municipal			Total
		IDMH 1	IDMH 2	IDMH 3	
		0,666 (0,500–0,715)*	0,758 (0,716–0,800)*	0,864 (0,801–0,970)*	0,762 (0,500–0,970)*
Características das UDH						
	Número de UDH (%)	3000 (33,4)	3030 (33,8)	2947 (32,8)	8977
	Número médio da população nas UDH (IC95%)	8811,3 (8452,9–9169,7)	8192,7 (7891,2–8494,2)	7446,6 (7141,0–7752,2)	8154,5 (7967,7; 8341,3)
	Área média das UDH por km^2^ (IC95%)	25,8 (15,6–35,9)	4,9 (3,3–6,5)	2,4 (2,1–2,8)	11,1 (7,6–14,5)
	Número médio de estabelecimentos de alimentos totais (IC95%)	19,0 (17,9–20,1)	34,3 (32,6–35,9)	75,7 (70,8–80,6)	42,8 (40,9–44,6)
Percentual médio de estabelecimentos de acordo com as categorias Locais-Nova						
	Percentual médio de locais fonte apenas de in natura† (IC95%)	22,1 (21,3–22,9)	25,4 (24,8–26,1)	29,6 (28,9–30,2)	25,7 (25,2–26,1)
	Percentual médio de locais fonte de in natura† (IC95%)	25,8 (25,0–26,7)	29,2 (28,5–29,8)	32,3 (31,6–32,9)	29,1 (28,6–29,5)
	Percentual médio de locais fonte de processados†(IC95%)	15,5 (14,8–16,2)	16,0 (15,4–16,6)	13,8 (13,3–14,3)	15,1 (14,8–15,5)
	Percentual médio de locais fonte de ultraprocessados†(IC95%)	67,5 (66,6–68,5)	64,2 (63,4–64,9)	63,1 (62,4–63,8)	64,9 (64,5–65,4)
	Percentual médio de locais fonte apenas de ultraprocessados†(IC95%)	58,6 (57,6–59,6)	54,8 (54,0–55,5)	53,9 (53,2–54,6)	55,8 (55,3–56,2)

* IDHM médio (intervalo de variação)

† Percentual=número de locais fonte*100/total de estabelecimentos varejistas de alimentos

UDH: Unidades de Desenvolvimento Humano; IDHM: Índice de Desenvolvimento Humano Municipal; IC: índice de confiança.

Fonte: Elaboração própria.

O Sudeste reúne o maior número de UDH (5.574) e as maiores densidades populacionais (população/área) e de estabelecimentos de alimentos totais (estabelecimentos totais/área). Em todas as regiões, observa-se aumento da densidade populacional e de locais de aquisição de alimentos do primeiro para o terceiro tercil de IDHM. A região Norte apresenta o menor número de UDH (395) e as maiores diferenças entre os tercis: no terceiro tercil de IDHM, a densidade de locais de aquisição de alimentos é 208 vezes maior e a densidade populacional, 40 vezes maior do que no primeiro tercil de IDHM. As capitais reúnem, em geral, menor área média, maior população e mais estabelecimentos de alimentos que as não capitais. Entre os extremos de IDHM, as capitais registram maior diferença de densidade populacional (5,4 vezes no 3º tercil de IDHM em relação ao 1º, contra 2,1 vezes nas não capitais), enquanto as não capitais apresentam maior diferença na densidade de estabelecimentos de alimentos (41 vezes no 3º tercil de IDHM em relação ao 1º, contra 25 vezes nas capitais) (Tabela 2). Ainda, em todas as regiões o percentual médio de locais fonte de ultraprocessados é superior ao de locais fonte de in natura (Tabela 3).

**Tabela 2:** Características das Unidades de Desenvolvimento Humano segundo macrorregiões brasileiras, capitais e não capitais, por tercis de Índice de Desenvolvimento Humano Municipal; 2023.

			IDMH 1	IDMH 2	IDMH 3	Total
			n (IC95%)	n (IC95%)	n (IC95%)	n (IC95%)
Número de UDH						
	Região						
		Sul	341	322	327	990
		Sudeste	1.897	1.852	1.825	5.574
		Centro-Oeste	280	210	205	623
		Nordeste	466	472	457	1.395
		Norte	134	130	131	395
	Situação						
		Capital	1.507	1.496	1.483	4.486
		Não capital	1.508	1.542	1.441	4.491
População média nas UDH						
	Região						
		Sul	6311,9 (5528,4–7095,3)	7458,7 (6592,0–8325,3)	7496,8 (6529,5–8464,2)	7076,3 (6571,2–7581,3)
		Sudeste	7020,5 (6673,6–7367,3)	8152,4 (7259,3–7982,1)	7620,7 (7259,3–7982,1)	7593,1 (7388,6–7797,6)
		Centro-Oeste	12570,3 (10607,4–14533,2)	8704,1 (7514,7–9893,5)	8204,4 (6410,0–9998,7)	9830,5 (8851,0–10809,9)
		Nordeste	13943,5 (12801,4–15085,5)	9268,9 (8363,9–10174,0)	7230,5 (6453,3–8007,8)	10162,7 (9592,2–10733,1)
		Norte	14019,8 (11481,2–16558,4)	10629,3 (8481,3–12777,3)	4714,3 (3653,8–5774,7)	9817,8 (8591,5–11044,1)
	Situação						
		Capital	10152,3 (9523,0–10781,5)	8008,7 (7578,7–8438,7)	8613,6 (8098,4–9128,8)	8928,8 (8620,7–9236,9)
		Não capital	7956,0(7565,7–8346,4)	8059,8; 7686,3–8433,2)	6265,4; 5947,6–6583,2)	7449,2 (7238,1–7660,3)
Área média das UDH por km^2^						
	Região						
		Sul	25,6 (10,2–40,9)	6,2 (4,7–7,8)	2,2 (1,8–2,6)	11,6 (6,2–16,9)
		Sudeste	8,9 (5,5–12,2)	4,8 (2,4–7,3)	2,1 (1,8–2,4)	5,3 (3,9–6,7)
		Centro-Oeste	66,4 (23,9–108,8)	8,5 (5,0–12,0)	6,9 (3,3–10,6)	27,3 (12,9–41,7)
		Nordeste	39,8 (24,6–55,0)	1,6 (1,4–1,8)	1,7 (1,3–2,1)	14,4 (9,2–19,6)
		Norte	156,4 (-45,7–358,5)	1,4 (1,1–1,8)	1,3 (0,8–1,8)	54,0 (-14,8–122,7)
	Situação						
		Capital	14,0 (-4,3–28,5)	2,4 (1,9–3,0)	2,2 (1,7–2,7)	6,3 (1,4–11,1)
		Não capital	36,0 (21,9–50,2)	8,3 (5,3–11,4)	2,9 (2,5–3,3)	15,9 (11,0–20,8)
Número médio de estabelecimentos de alimentos totais						
	Região						
		Sul	17,4 (14,5–20,4)	39,0 (33,7–44,2)	87,5 (72,4–102,6)	47,6 (41,9–53,3)
		Sudeste	14,0 (13,0–15,0)	32,4 (30,5–34,4)	75,5 (69,6–81,4)	40,3 (38,1–42,4)
		Centro-Oeste	43,1 (34,5–51,7)	48,5 (40,5–56,6)	99,4 (68,6–130,3)	63,5 (52,4–74,5)
		Nordeste	29,2 (25,2–33,2)	34,3 (30,1–38,6)	67,0 (56,3–77,6)	43,3 (39,2–47,4)
		Norte	22,5 (17,2–27,7)	30,2 (23,6–36,7)	40,7 (2,3–52,2)	31,1 (26,3–35,8)
	Situação						
		Capital	23,3 (21,4–25,1)	39,0 (26,2–41,8)	93,4 (84,8–102,0)	51,7 (48,5–57,9)
		Não capital	17,0 (15,4–18,5)	29,2 (27,4–31,1)	56,4 (52,1–60,7)	33,8 (32,1–35,5)

IDHM: Índice de Desenvolvimento Humano Municipal; IC: índice de confiança; UDH: Unidades de Desenvolvimento Humano.

Fonte: Elaboração própria.

**Tabela 3:** Percentual médio de locais de aquisição de alimentos das categorias Locais-Nova em relação ao total de locais de aquisição de alimentos, por macrorregiões brasileiras e tercis de Índice de Desenvolvimento Humano Municipal; 2023.

		Percentual médio de locais fonte apenas de in natura* % (IC95%)	Percentual médio de locais fonte de in natura* % (IC95%)	Percentual médio de locais fonte de processados* % (IC95%)	Percentual médio de locais fonte de ultraprocessados* % (IC95%)	Percentual médio de locais fonte apenas de ultraprocessados* % (IC95%)
Sul						
	Total	23,2 (22,0–24,5)	27,8 (26,5–29,1)	9,9 (9,0–10,8)	75,8 (74,5–77,1)	61,8 (60,4–63,2)
	1º tercil IDHM	15,0 (12,8–17,1)†	20,8 (18,4–23,1)†	10,2 (8,4–12,1)	83,9 (81,6–86,2)†	68,7 (66,0–71,3)†
	2º tercil IDHM	24,0 (21,0–26,0)	28,5 (26,5–30,5)	11,2 (9,6–12,7)	75,1 (73,1–77,1)	59,6 (57,5–61,8)
	3º tercil IDHM	31,1 (29,0–33,1)	34,3 (32,3–36,3)	8,2 (7,0–9,4)	68,1 (66,0–70,2)	56,7 (54,6–58,9)
Sudeste						
	Total	25,0 (24,5–25,5)	28,6 (28,1–29,2)	17,3 (16,9–17,8)	63,2 (62,6–63,8)	54,1 (53,4–54,7)
	1º tercil IDHM	22,4 (21,4–23,5)†	26,3 (25,2–27,4)†	17,9 (16,9–18,8)‡	64,8 (63,6–66,0)‡	55,8 (54,6–57,1)†
	2º tercil IDHM	24,8 (24,0–25,6)	28,9 (28,0–29,8)	17,9 (17,1–18,6)	62,6 (61,7–63,5)	53,2 (52,3–54,2)
	3º tercil IDHM	27,9 (27,1–28,7)	30,7 (29,9–31,5)	16,3 (15,6–16,9)	62,3 (61,4–63,1)	53,0 (52,1–53,9)
Centro-Oeste						
	Total	27,6 (26,3–28,9)	35,2 (33,9–36,6)	10,9 (10,0–11,7)	72,4 (71,1–73,7)	53,9 (52,5–55,3)
	1º tercil IDHM	22,2 (20,0–24,3)†	33,0 (30,5–35,4)‡	10,6 (9,1–12,2)	77,8 (75,7–80,0)†	56,4 (53,8–59,0)
	2º tercil IDHM	29,1 (26,9–31,5)	36,3 (34,1–38,6)	11,3 (10,1–12,6)	70,8 (68,5–73,1)	52,3 (50,0–54,6)
	3º tercil IDHM	31,5 (29,5–33,5)	36,3 (34.2–38,5)	10,6 (8,9–12,3)	68,5 (66,5–70,5)	53,1 (50,9–55,3)
Nordeste						
	Total	28,9 (27,9–30,0)	28,9 (27,9–30,0)	12,7 (11,9–13,5)	58,3 (57,2–59,5)	58,4 (57,2–59,5)
	1º tercil IDHM	25,2 (23,3–27,2)†	25,2 (23,3–27,2)†	13,6 (12,2–15,4)†	61,1 (59,0–63,3)‡	61,1 (59,0–63,2)‡
	2º tercil IDHM	27,9 (26,1–29,7)	27,9 (26,1–29,7)	14,5 (13,1–16,0)	57,6 (55,5–59,7)	57,6 (55,5–59,7)
	3º tercil IDHM	33,8 (32,0–35,6)	33,8 (32,0–35,6)	9,9 (8,8–11,0)	56,3 (54,4–58,2)	56,3 (54,4–58,2)
Norte						
	Total	26,7 (24,5–28,8)	29,4 (27,1–31,6)	12,2 (10,8–13,6)	73,4 (71,2–75,5)	58,4 (56,1–60,8)
	1º tercil IDHM	22,3 (18,2–26,5)†	24,7 (20,4–28,9)†	13,0 (10,1–16,0)	77,7 (73,5–81,8)†	62,3 (57,7–65,9)‡
	2º tercil IDHM	25,6 (22,4–28,8)	29,2 (25,9–32,5)	11,8 (9,9–13,7)	74,4 (71,2–77,6)	59,0 (55,6–62,5)
	3º tercil IDHM	32,1 (28,4–35,9)	34,4 (30,6–38,1)	11,7 (9,3–14,1)	67,9 (64,1–71,6)	53,9 (50,1–57,7)

* Percentual=número de locais fonte*100/total de estabelecimentos varejistas de alimentos;

† p de tendência <0,01;

‡ p de tendência <0,05.

IDHM: Índice de Desenvolvimento Humano Municipal; IC: índice de confiança; UDH: Unidades de Desenvolvimento Humano.

Fonte: Elaboração própria.

Na região Sul, as diferenças entre os tercis de IDHM são expressivas. O percentual de locais fonte apenas de in natura no primeiro tercil de IDHM corresponde à metade do observado no terceiro. O Sudeste apresenta o maior percentual de locais fonte de processados (17,3%), enquanto o Centro-Oeste se destaca pela maior proporção de locais fonte de in natura (35,2%) e o Nordeste pelo menor percentual de locais fonte de ultraprocessados (58,3%). No Norte, as diferenças entre os tercis de IDHM são relativamente homogêneas, exceto para locais fonte de processados, que não variam.

As capitais concentram 4% mais locais fonte apenas de ultraprocessados e 6% mais locais fonte apenas de in natura. A maior diferença entre os tercis ocorre nos locais fonte apenas de in natura, favorecendo as UDH de maior IDHM (8,4% de variação nas capitais e 6,5% nas não capitais) (Tabela 4).

**Tabela 4:** Percentual médio de locais de aquisição de alimentos das categorias Locais-Nova, em relação ao total de locais de aquisição de alimentos, por Unidades de Desenvolvimento Humano, capitais e não capitais e tercis de Índice de Desenvolvimento Humano Municipal; 2023.

		Percentual médio de locais fonte apenas de in natura*	Percentual médio de locais de in natura*	Percentual médio de locais fonte de processados*	Percentual médio de locais fonte de ultraprocessados*	Percentual médio de locais fonte apenas de ultraprocessados*
		% (IC95%)	% (IC95%)	% (IC95%)	% (IC95%)	% (IC95%)
Capital						
	Total	26,4 (25,8–27,0)	28,9 (28,3–29,5)	14,1 (13,6–14,6)	65,3 (64,7–65,9)	56,9 (56,2–57,5)
	1º tercil IDHM	22,4 (21,2–23,5)†	25,0 (23,8–26,2)†	14,9 (13,9–15,9)†	67,8 (66,5–69,1)†	60,1 (58,7–61,4)†
	2º tercil IDHM	26,1 (25,2–27,0)	29,0 (28,1–30,0)	14,6 (13,9–15,4)	65,2 (64,1–66,2)	56,3 (55,3–57,4)
	3º tercil IDHM	30,8 (29,9–31,7)	32,9 (32,0–33,8)	12,8 (12,1–13,5)	62,9 (61,9–63,8)	54,2 (53,2–55,2)
Não capital						
	Total	24,9 (24,4–25,5)	29,2 (28,6–29,8)	16,1 (15,6–16,6)	64,6 (63,9–65,2)	54,6 (54,0–55,3)
	1º tercil IDHM	21,8 (20,7–22,9)†	26,3 (25,1–27,5)†	15,8 (14,8–16,7)	67,5 (66,2–68,9)†	57,9 (56,5–59,3)†
	2º tercil IDHM	24,9 (24,0–25,8)	29,6 (28,6–30,5)	17,0 (16,2–17,9)	63,6 (62,6–64,7)	53,3 (52,2–54,4)
	3º tercil IDHM	28,3 (27,3–29,2)	31,9 (30,9–32,8)	15,5 (14,7–16,2)	62,5 (61,5–63,5)	52,6 (51,6–53,6)

IC: índice de confiança; IDHM: Índice de Desenvolvimento Humano Municipal.

* Percentual=número de locais fonte*100/total de estabelecimentos varejistas de alimentos;

† p de tendência <0,01.

Fonte: Elaboração própria.

Os mapas apresentam a distribuição espacial do indicador de saudabilidade do ambiente alimentar (razão entre locais de aquisição fonte de ultraprocessados e locais fonte apenas de in natura). Em todas as regiões metropolitanas e regiões integradas de desenvolvimento econômico predomina a cor rosa, indicando predominância de locais fonte de ultraprocessados nas UDH. A cor rosa brilhante, que representa maior predominância desses locais no terceiro tercil de IDHM, concentra-se nos centros urbanos e nas áreas litorâneas das regiões metropolitanas. O mapa da RM de São Paulo, com maior população e número de estabelecimentos de alimentos, é apresentado na Figura 1. Os mapas das demais regiões estão disponíveis no material suplementar (Figuras Suplementares 1 a 23). Poucas UDH apresentam proporção equilibrada entre as duas categorias. Na Baixada Santista, Belém, Belo Horizonte, Maceió, Rio de Janeiro, São Paulo, Sorocaba, Vale do Paraíba, Vale do Rio Cuiabá e Vitória, a proporção de UDH com predominância de locais fonte de ultraprocessados cresce com o aumento do tercil de IDHM. Nas demais regiões, observa-se o padrão inverso, com exceção do Distrito Federal e de Porto Alegre, onde nenhum desses padrões foi observado.

**Figura 1:** Distribuição espacial do indicador de saudabilidade do ambiente alimentar (razão entre locais fonte apenas de in natura e minimamente processados e fonte de ultraprocessados) na Região Metropolitana de São Paulo.

Fonte: Elaboração própria.

## DISCUSSÃO

Observaram-se diferenças socioeconômicas na distribuição espacial dos estabelecimentos de alimentos. Áreas de menor renda têm menor concentração populacional e disponibilidade de estabelecimentos, tanto em densidade territorial quanto em número relativo à população da UDH.

Em países como China, Singapura e Reino Unido, regiões com menor nível socioeconômico apresentam maior densidade de estabelecimentos de alimentos^31,32^. Já no México e no Brasil, áreas socioeconomicamente desfavorecidas exibem menor concentração desses estabelecimentos^33-36^, provavelmente em razão de contextos socioeconômicos e padrões de desenvolvimento distintos de países de alta renda.

No Norte Global, a maior densidade populacional em regiões menos desenvolvidas atrai estabelecimentos pela maior demanda^31^. Em países em desenvolvimento, a precariedade da infraestrutura urbana em áreas vulneráveis favorece a expansão do mercado informal, o que pode explicar a menor concentração de estabelecimentos no primeiro tercil de IDHM. Essas regiões foram mais afetadas pela pandemia de covid-19, com fechamento de estabelecimentos que não reabriram^36,37^. No Rio Grande do Sul, os minimercados apresentaram maior redução entre 2010 e 2022^36^, sendo mais frequentes em áreas socioeconomicamente desfavorecidas^38^. Já no Reino Unido, houve aumento de estabelecimentos de delivery de alimentos em áreas desfavorecidas e redução nas favorecidas no período pós-pandemia^39^.

Os estabelecimentos fonte de ultraprocessados correspondem a quase dois terços do total e se concentram no primeiro tercil de IDHM. Já os estabelecimentos fonte de in natura representam menos de um terço do total dos estabelecimentos de alimentos e se concentram no terceiro tercil de IDHM. Assim, residentes de regiões menos favorecidas enfrentam maiores barreiras para adotar as recomendações do Guia Alimentar para a População Brasileira, cuja regra de ouro é "prefira sempre alimentos in natura e minimamente processados e preparações culinárias a alimentos ultraprocessados"^29^.

O Sudeste apresenta a menor diferença entre os tercis de IDHM, indicando exposições a ambientes alimentares semelhantes, ainda que em contextos sociais distintos. Também concentram o maior percentual médio de locais fonte de processados. Na região, as padarias são classificadas como fonte de processados, enquanto nas demais regiões são também fonte de ultraprocessados. Em São Paulo, região metropolitana com o maior número de UDH, as padarias são centrais na cultura alimentar local, o que pode explicar o maior percentual de locais fonte de processados no Sudeste.

Na região Sul, 83,9% dos estabelecimentos do primeiro tercil de IDHM são fontes de ultraprocessados, indicando maior exposição a ambientes alimentares não saudáveis em áreas menos favorecidas, em comparação às demais regiões e tercis de IDHM. Um estudo que estimou a participação calórica de ultraprocessados nos municípios brasileiros identificou maiores médias de consumo na região Sul^40^. Embora realizados em períodos distintos e com diferentes fontes de dados, os achados convergem e evidenciam a coerência entre disponibilidade e consumo de ultraprocessados na região.

O Guia Alimentar para a População Brasileira recomenda evitar a compra em locais que comercializam exclusivamente alimentos ultraprocessados como estratégia para superar obstáculos relacionados à oferta de alimentos^29^. A presença de ultraprocessados no ambiente pode dificultar escolhas alimentares mais saudáveis, mesmo quando alimentos in natura e minimamente processados estão disponíveis.

Essa dinâmica pode ser explicada pelos determinantes comerciais da saúde, entendidos como estratégias adotadas por atores comerciais para ampliar mercados e influenciar comportamentos e condições de saúde^7^. No caso dos ultraprocessados, incluem práticas de marketing e organização do varejo que favorecem sua disponibilidade e visibilidade nos ambientes alimentares^41,42^, conferindo vantagem competitiva em relação aos alimentos in natura e minimamente processados. Os supermercados, fonte simultânea de alimentos ultraprocessados e in natura, exemplificam esse processo. Pequenos produtores e agricultores familiares enfrentam dificuldades para inserir sua produção nesses locais, devido a contratos que incluem prazos longos de pagamento, venda consignada e exigências de padronizações de tamanho e formato de hortaliças e frutas. Em contrapartida, grandes corporações de ultraprocessados possuem recursos para adquirir melhores espaços nas prateleiras^41^, um dos principais facilitadores de venda no varejo^43-46^. Esse favorecimento se reflete no padrão de compras da população, com maior participação de ultraprocessados nas compras realizadas em supermercados^47^.

Ao contrário do esperado, não foi observada grande diferença entre as capitais e não capitais para nenhuma das categorias de locais de aquisição. Esse resultado pode ser explicado pela seleção de municípios localizados em regiões metropolitanas, que podem apresentar características semelhantes às capitais, como população média, grau de urbanização e renda.

A utilização dos mapas permite identificar com objetividade os territórios mais vulneráveis, que devem ser priorizados em políticas públicas voltadas à transformação do ambiente alimentar, configurando-se como uma importante ferramenta estratégica de apoio à gestão pública.

A criação de ambientes alimentares saudáveis é fundamental para promover a alimentação saudável e requer ações intersetoriais entre os setores de saúde e abastecimento. Programas, políticas e ações devem favorecer escolhas mais saudáveis, como o incentivo à abertura de estabelecimentos fonte apenas de alimentos in natura e minimamente processados em áreas com baixa disponibilidade desses alimentos^48^.

O estudo possui limitações inerentes ao delineamento ecológico. A presença de locais fonte de aquisição de alimentos não necessariamente reflete o comportamento real de compra dos indivíduos. Os resultados podem ser influenciados pelo Problema de Unidade de Área Modificável (MAUP, acrônimo em inglês), relacionado a vieses decorrentes do agrupamento das unidades espaciais de análise^49^, especialmente em UDH mais extensas, onde diferentes padrões de distribuição dos estabelecimentos podem coexistir e ser representados por uma única média.

Outra limitação refere-se ao uso de diferentes bases de dados secundárias, que podem apresentar variações na cobertura, atualização e qualidade das informações. Ainda assim, essas bases permitiram analisar a distribuição dos estabelecimentos em ampla cobertura territorial e identificar padrões espaciais do ambiente alimentar em diferentes contextos socioeconômicos.

O autopreenchimento da RAIS pelos proprietários dos estabelecimentos pode gerar erros na classificação da atividade econômica. Além disso, pela própria natureza da base de dados, foi possível avaliar apenas estabelecimentos formais de alimentos. Dados sobre feiras livres e outros equipamentos de segurança alimentar e nutricional não estão disponíveis para todas as cidades avaliadas. No entanto, a utilização de uma base de dados secundária com informações de todos os estabelecimentos formais de alimentos é o que permite um estudo dessa magnitude.

O georreferenciamento com base no ponto médio do CEP é outra limitação, entretanto, o CEP é o dado disponível mais preciso para a localização dos estabelecimentos e uma medida amplamente utilizada em estudos sobre o ambiente alimentar^50^.

Este estudo avaliou a disponibilidade de estabelecimentos de alimentos, sem considerar o tempo e o modo de deslocamento necessários para acessá-los ou os preços praticados. A análise foi restrita às regiões metropolitanas, limitando a generalização dos achados para outros contextos.

Por fim, há defasagem temporal entre os dados da RAIS de 2023 e as informações socioeconômicas do Censo Demográfico de 2010. As Unidades de Desenvolvimento Humano possuem indicadores socioeconômicos calculados apenas para esse Censo, sem estimativas mais recentes com a mesma desagregação territorial.

Entre as fortalezas do estudo, destaca-se sua abrangência, pois se trata da mais ampla descrição do ambiente alimentar já realizada no Brasil, contemplando 24 RM das 5 macrorregiões. O uso da metodologia Locais-Nova constitui outro ponto forte, por estar ancorada na realidade do consumo e aquisição de alimentos do país.

Os achados fornecem subsídios para a elaboração de políticas públicas de abastecimento e promoção da alimentação adequada e saudável e reforçam a necessidade de estratégias que considerem desigualdades regionais na oferta de alimentos, orientando ações de apoio à abertura e manutenção de locais fonte apenas de alimentos in natura e minimamente processados, além da regulação de locais fonte de ultraprocessados. Essas medidas são especialmente importantes em áreas com predominância de ultraprocessados, que dificultam escolhas saudáveis.

## Material Suplementar

Material Suplementar
